# Age-associated B cells predict coronary events in humans and aggravate murine atherosclerosis

**DOI:** 10.1093/cvr/cvag154

**Published:** 2026-07-13

**Authors:** Jill de Mol, Pernilla Katra, Virginia Smit, Anna Witteveen, Lukas Tomas, Linda Andersson, Scott W M Engels, Samuel Andersson, Mireia N A Bernabé Kleijn, Alexandru Schiopu, Eva Bengtsson, Ilze Bot, Daniel Engelbertsen, Johan Kuiper, Harry Björkbacka, Amanda C Foks

**Affiliations:** Division of BioTherapeutics, Leiden Academic Centre for Drug Research (LACDR), Leiden University, Einsteinweg 55, Leiden 2333CC, The Netherlands; Department of Clinical Sciences Malmö, Lund University, Malmö, Sweden; Division of BioTherapeutics, Leiden Academic Centre for Drug Research (LACDR), Leiden University, Einsteinweg 55, Leiden 2333CC, The Netherlands; Division of BioTherapeutics, Leiden Academic Centre for Drug Research (LACDR), Leiden University, Einsteinweg 55, Leiden 2333CC, The Netherlands; Department of Medicine, LMU University Hospital, Munich, Germany; German Centre for Cardiovascular Research (DZHK), partner site Munich Heart Alliance, Munich, Germany; Department of Clinical Sciences Malmö, Lund University, Malmö, Sweden; Division of BioTherapeutics, Leiden Academic Centre for Drug Research (LACDR), Leiden University, Einsteinweg 55, Leiden 2333CC, The Netherlands; Department of Clinical Sciences Malmö, Lund University, Malmö, Sweden; Division of BioTherapeutics, Leiden Academic Centre for Drug Research (LACDR), Leiden University, Einsteinweg 55, Leiden 2333CC, The Netherlands; Department of Translational Medicine, Lund University, Malmö, Sweden; Department of Internal Medicine, Skåne University Hospital, Lund, Sweden; Nicolae Simionescu Institute of Cellular Biology and Pathology, Bucharest, Romania; Department of Clinical Sciences Malmö, Lund University, Malmö, Sweden; Department of Biomedical Science, Malmö University, Malmö, Sweden; Biofilms—Research Center for Biointerfaces, Malmö University, Malmö, Sweden; Division of BioTherapeutics, Leiden Academic Centre for Drug Research (LACDR), Leiden University, Einsteinweg 55, Leiden 2333CC, The Netherlands; Department of Clinical Sciences Malmö, Lund University, Malmö, Sweden; Division of BioTherapeutics, Leiden Academic Centre for Drug Research (LACDR), Leiden University, Einsteinweg 55, Leiden 2333CC, The Netherlands; Department of Clinical Sciences Malmö, Lund University, Malmö, Sweden; Division of BioTherapeutics, Leiden Academic Centre for Drug Research (LACDR), Leiden University, Einsteinweg 55, Leiden 2333CC, The Netherlands

**Keywords:** Age-associated B cells, Aging, Atherosclerosis, Cardiovascular disease

## Abstract

**Aims:**

Aging is a major risk factor for cardiovascular disease (CVD) and is also linked to a functional decline of the immune system. B cells contribute to atherosclerosis progression by antibody secretion, antigen presentation and T-cell regulation. During aging, CD11c^+^CD21^low^ B cells accumulate, but it is unclear whether these age-associated B cells (ABCs) are linked with incident cardiovascular events in humans and if they contribute to the progression of atherosclerosis.

**Methods and results:**

To investigate associations with first-time coronary events (CEs), circulating B cells were phenotyped with flow cytometry in a case–control study (*N* = 604) nested in the Malmö Diet and Cancer study cohort with a median of 14 years follow-up. Splenic B cells in young (5 months) and aged (21 months) female *Ldlr*^−/−^ mice were characterized with single-cell RNA-sequencing coupled with B-cell receptor (BCR)-sequencing. Atherogenicity of ABCs was determined by adoptive transfer to *Ldlr^−/−^* and *Ldlr*^−/−^*Rag1*^−/−^ mice. Compared with other B-cell populations, ABCs showed a significant positive correlation with age (Spearman’s rho 0.24; *P* < 0.0001; across 46–68 years of age) and accumulated in aged atherosclerotic mice. CE cases had higher counts of ABCs than controls and regression analysis revealed an association with incident CEs, that was independent of cardiovascular risk factors [odds ratio: 1.92 (95% confidence interval 1.13–3.27), *P* = 0.016, comparing the fourth vs. first quartile]. Adoptive transfer of ABCs to recipient mice promoted atherosclerotic lesion development. In *Ldlr^−/−^ Rag1^−/−^* mice, ABCs also increased necrotic cores (*P* < 0.05). B cells recovered from recipient mice after transfer expressed CD138 and secreted antibodies. In line with these findings, we demonstrated that ABCs expressed plasma cell (PC) differentiation genes and showed the greatest clonal expansion within the B-cell compartment, with extensive clonal overlap with PCs.

**Conclusion:**

We show that ABCs predict first-time CEs in humans and contribute to the immunopathology of atherosclerosis in mice, indicating that clonal expansion and accumulation of ABCs contribute to the strong association between age and CVD. ABCs may serve as potential prognostic and therapeutic targets for CVD.

## Graphical Abstract

**Graphical Abstract:**
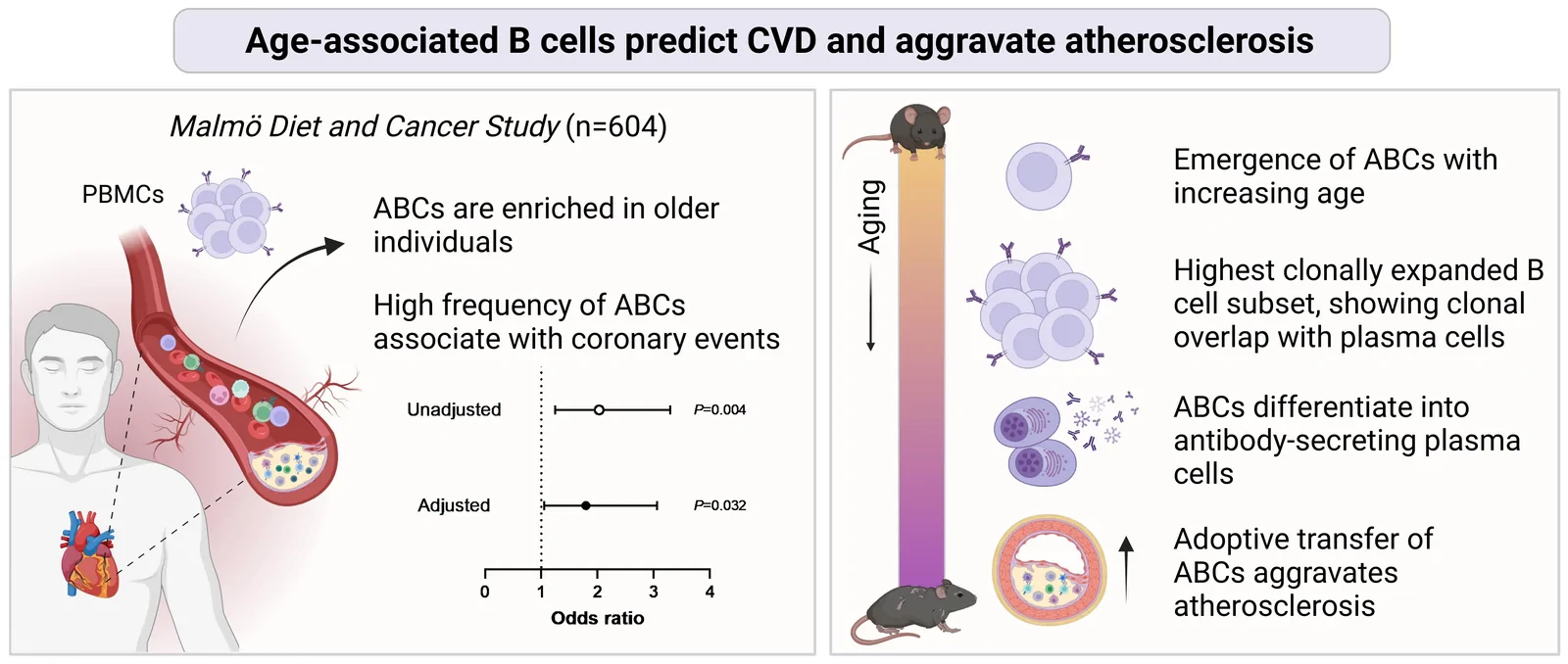

**Time for primary review: 66 days**

## 1. Introduction

Aging leads to a gradual dysfunction of the immune system, which is accompanied by increased prevalence of inflammatory diseases, including cardiovascular disease (CVD).^1,2^ Aging is also associated with detrimental changes in B-cell immunity.^3^ Fewer circulating naïve B cells are observed with age, due to reduced levels of early progenitor B cells in the bone marrow (BM)^4^ and B-cell expansion is impaired due to age-related dysfunction of CD4^+^ T helper cells.^5^ Moreover, aging has been shown to lead to a reduction in antibody affinity and neutralization capacity, and increased autoantibody production.^6,7^ These age-related changes in B-cell immunity have been linked to the pathogenesis of infectious and autoimmune diseases.^3^ We and others have shown that some B-cell populations are associated with CVD in humans.^8–11^ Experimental and human studies have demonstrated that B cells affect atherosclerosis in a subset-specific manner, with B1-like and regulatory B cells generally exerting atheroprotective functions through secretion of natural IgM and IL-10, whereas conventional follicular B2 cells (FOBs) and certain switched memory (SM) B-cell populations promote lesion development by supporting pro-inflammatory T helper responses and producing class-switched antibodies against modified lipoproteins.^3,12^ In line with this, depletion or adoptive transfer experiments in atherosclerosis-prone mice have revealed that total B-cell depletion can aggravate disease when protective B1/Breg compartments are lost, while selective targeting of pro-atherogenic B2 cells attenuates plaque burden, supporting a nuanced, subset-dependent contribution of B cells to atherogenesis.^13–16^ Human studies further indicate that circulating B-cell activation pathways are enriched in patients with coronary artery disease, and that frequencies of distinct B-cell subsets, including CXCR4-expressing putative human B1 cells and memory B cells, associate with clinical CVD and plaque phenotype, underscoring the clinical relevance of B-cell heterogeneity in atherosclerosis.^9,17,18^ Still, how age-associated changes in B-cell immunity affect CVD remains poorly understood.

The main pathology underlying CVD is atherosclerosis, which is characterized by formation of plaques rich in lipids and immune cells in the arterial wall.^19,20^ Although aging leads to immunosenescence and exhaustion that modify the adaptive immune responses over time, most mechanistic studies of atherosclerosis have been performed in young mice, with a focus on the role of T cells and less so on that of B cells. We and others have, however, recently identified and described the presence of age-associated B cells (ABCs) in atherosclerotic aortas of aged *Ldlr^−/−^* and *Apo*e*^−/−^* mice as well as in plaques from patients undergoing carotid endarterectomy.^21–23^ ABCs are antigen-experienced B cells that can be characterized by the expression of markers including CD11c and low levels of CD21 on the cell surface.^24^ CD11c^+^ ABCs accumulate in plaques of aged atherosclerotic mice, where they display enhanced expression of T-bet, co-stimulatory molecules and genes involved in antigen presentation.^21^ In addition, CD11c^+^ ABCs are superior antigen-presenting cells compared with FOBs, thereby potentiating T-cell activation. In both mouse models and human cohorts, circulating and plaque-resident ABCs increase with age and higher frequencies of these CD11c^+^ ABCs correlate with greater atherosclerotic burden, suggesting a link between ABC expansion and disease progression.^21,22^ Upon activation, ABCs secrete pro-inflammatory cytokines [e.g. tumour necrosis factor α (TNFα)] and high levels of autoantibodies, which could contribute to the autoimmune mechanisms part of the atherosclerosis aetiology.^25^ Together with broader age-related remodelling of the B-cell compartment—including contraction of naïve B cells, accumulation of late/exhausted memory and double-negative B cells, and qualitative changes in antibody repertoires^26^—these findings support the concept that B-cell aging, and ABC expansion in particular, may critically shape vascular inflammation and atherogenesis, but the precise causal mechanisms and therapeutic potential of targeting ABCs in CVD remain to be fully elucidated.

In the present study, we address the knowledge gaps in age-related B-cell immunity in CVD and atherosclerosis by determining associations between ABCs and incident first-time coronary events (CEs) in a general population-based cohort and investigating the atherogenicity of adoptively transferred ABCs in mice.

## 2. Methods

A more detailed version of the methods can be found in the Supplementary material.

### 2.1 Human cohort study design

A case–control study was nested within the population-based Malmö Diet and Cancer (MDC) cohort by matching 302 CE cases to 302 controls by incidence density sampling of CE–free control participants of the same age and sex (see Supplementary material online, *Figure S1*). The study design, methods, baseline data collection and flow cytometry data acquisition are detailed in the Supplementary material and as described previously.^27^ The study was approved by the regional ethics review board (LU 51-90 and LU 532-2006) and was conducted in accordance with the Declaration of Helsinki. All participants gave written informed consent.

### 2.2 Animals

All animal work was approved by local animal ethics committees and executed in line with governmental guidelines and the Directive 2010/63/EU of the European Parliament on the protection of animals used for scientific purposes. Female *Ldlr^−/−^* and *Ldlr^−/−^ Rag1^−/−^* mice (young: 2–5 months, aged: 18–22 months) on a C57BL/6J genetic background were bred and aged in-house. *Tcrα^−/−^* (B6.129S2-Tcratm1Mom/J) mice were purchased from Jackson Laboratories and crossed with *Ldlr^−/−^* mice in-house. Female *Tcrα^−/−^ Ldlr^−/−^* mice were aged for 44–51 weeks. At the end of experiment, mice were anesthetized by a subcutaneous injection containing a cocktail of ketamine (100 mg/kg), atropine (0.5 mg/kg) and xylazine (10 mg/kg) and mice were sacrificed by retro-orbital bleeding. One mouse was excluded from the experiment due to hydronephrosis.

### 2.3 Single-cell RNA sequencing of CD45^+^ splenocytes

Single-cell splenocyte suspensions were obtained from chow diet (CD)-fed female *Ldlr^−/−^* mice (young: 5 months, aged: 21 months), blocked with Mouse TruStain FcX™ and stained with Fixable Viability Dye-eFluor 780, CD45-PE, and TotalSeq-C antibodies (see Supplementary material online, *Tables S2* and *S3*). Alive CD45^+^ cells were sorted (see Supplementary material online, *Figure S3*) and loaded into a 10x Chromium Single Cell Controller (10x Genomics) along with gel beads, partitioning oil, and reverse transcription reagents to generate single-cell gel bead-in-emulsions (GEMs). ScRNA-seq and scBCR-seq libraries were prepared using the Next GEM Single Cell 5′ Kit V2 and Single Cell V(D)J Enrichment Kit, Mouse B-cell (10x Genomics). All samples were sequenced with an Illumina HiSeq2500 sequencer to achieve library construction.

### 2.4 Adoptive transfer of ABCs

Alive, singlet ABCs (CD19^+^ CD23^−^ CD11b^+^/CD11c^+^) from CD-fed female *Ldlr^−/−^* mice (18–21 months old) were sorted using a FACS Aria II SORP (BD Biosciences) for adoptive transfer into female *Ldlr^−/−^* (*n* = 8/group) or *Ldlr^−/−^Rag1^−/−^* mice (*n* = 13–14/group) (2–3 months old). Mice received intravenous injections of 5 × 10^5^ FACS-sorted ABCs or phosphate buffered saline (control) at *T* = 0 and *T* = 4 weeks and were fed a western diet (WD) for 8 weeks.

### 2.5 Statistics

Statistical analyses on human data were performed in IBM SPSS Statistics 27. Figures were made in GraphPad Prism 9.1 and Cluster Explorer (FlowJo 10.10.0). Baseline data is presented as median with interquartile range (IQR) if continuous or count (percent of the total) if categorical. Differences in baseline measurements between cases and controls were analysed with the Mann–Whitney or χ^2^ test. Associations between B-cell populations (cell counts divided into quartiles) and incident CE were explored using conditional logistic regression conditioned for the matched case–control pairs. Quartile 1 with the lowest values of B cells was used as the reference. The regression models (matched for age and sex) were unadjusted or adjusted for CVD risk factors [total cholesterol, HDL, diabetes, systolic blood pressure (BP), and current smoking]. HDL values were natural logarithm transformed due to their non-normal distribution.

Data of the murine experiments are reported as mean ± standard error of the mean. Outliers were identified and removed using Grubbs outlier test (*a* = 0.05). Normally distributed data of two groups were tested for significance using an unpaired *t*-test. Data from more than two groups were analysed by a one-way analysis of variance (ANOVA). If data were not normally distributed, two groups were compared using a Mann–Whitney test, and three or more groups were compared via a Kruskal–Wallis test. *P*-values of <0.05 were considered significant. Statistical analyses were performed using GraphPad Prism 9.0 (GraphPad Software).

## 3. Results

### 3.1 Human ABCs are enriched in older individuals

**Figure 1:**
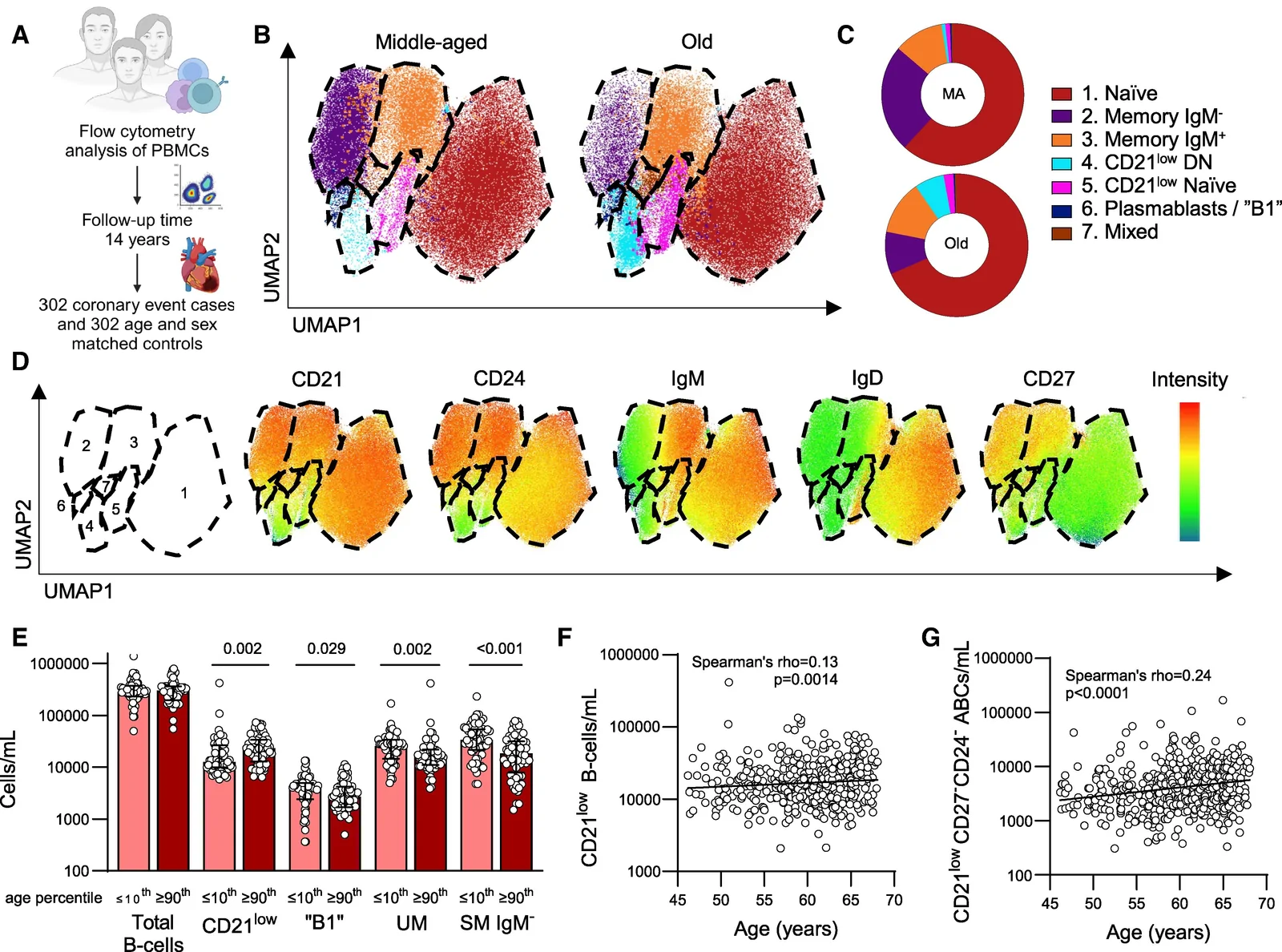
ABCs accumulate with increasing age in humans. (*A*) Design of the MDC study. (*B*) Unsupervised clustering combined with UMAP visualization of major B-cell populations in concatenated flow cytometry data from 4 old (≥66 years old) and 4 middle-aged (≤50 years old) individuals. (*C*) Proportions of each major B-cell cluster in middle-aged (MA) and old individuals. (*D*) Expression of B-cell markers visualized on the UMAP. (*E*) Number of total B cells, CD21^low^, ‘B1’, IgD^+^CD27^+^ UM and IgM^−^IgD^−^CD27^+^ SM (IgM^−^ SM) B cells in the youngest 10% (≤10th age percentile, *N* = 60) and the oldest 10% (≥90th age percentile, *N* = 60). One value could not be plotted on the log scale. (*F*) Number of CD21^low^ B cells per mL blood vs. age (*N* = 604). (*G*) Number of CD21^low^CD27^−^CD24^−^ ABCs per mL blood vs. age (*N* = 604). Data is presented as individual values. Group data are presented with median and IQR and analysed by Mann–Whitney tests. Correlation data are shown with a linear trend line and significance determined using Spearman’s rank test. Graphical elements were created with biorender.com.

To explore associations between B-cell populations, age and incident first-time CEs, we immunophenotyped cryopreserved peripheral blood mononuclear cells collected at the study baseline in 604 individuals from the population-based MDC study (*Figure 1A*). Individuals who suffered an incident CE (cases) during 14-years median follow up (*N* = 302) were matched 1:1 based on age and sex to incident-free individuals (controls), forming a nested case–control cohort of 226 women and 378 men with a median age of 61 (IQR 57–64) years at the time of inclusion. Based on the expression pattern of surface markers in Uniform Manifold Approximation and Projection (UMAP) analysis of 8 representative individuals, B cells could be mapped into 7 main subpopulations (*Figure 1B–D*, Supplementary material online, *Figure*  *S4*) that were further gated into a total of 20 defined B-cell populations for all 604 individuals (see Supplementary material online, *Figure S2*). Two of the mapped B-cell subpopulations, clusters 4 and 5, were characterized by low CD21 expression and these CD21^low^ B cells were markedly more pronounced in older compared with middle-aged individuals (*Figure 1B* and *C*). In contrast, the cluster containing IgM^−^IgD^−^CD27^+^ SM (IgM^−^ SM), cluster 2, was less pronounced in older individuals. With further analysis comparing the youngest 10% of the individuals (age range 46.2–51.9 years) to the oldest 10% (age range 65.6–68.1 years) in the cohort, we found that the CD21^low^ B-cell subset was the only subset that was significantly increased in the oldest individuals, almost two-fold, (*P* = 0.002, *Figure 1E*). Additionally, the CD43^+^CD27^+^ (‘B1’), IgD^+^CD27^+^ unswitched memory (UM) and IgM^−^ SM B cells were decreased in the oldest individuals compared with the youngest (*P* = 0.029, *P* = 0.002 and *P* < 0.001, respectively, *Figure 1E*). The increase in numbers of CD21^low^ and decreases in numbers of ‘B1’, UM and IgM^−^ SM B cells with age were confirmed by correlation analyses including all study participants (*Figure 1F*, Supplementary material online, *Table S5*). There was no difference in total B-cell counts between the oldest and youngest individuals, *P* = 0.996, nor any correlation with age, *P* = 0.16 (*Figure 1E*, Supplementary material online, *Table S5*). A subset of the CD21^low^ B cells that increased with age were also characterized by low expression of CD27 and CD24 (*Figure 1D*, Supplementary material online, *Figure S2*). The low expression of CD21, CD27, and CD24 coincides with CD11c expression, identifying these cells as ABCs (Supplementary material online, *Figure S5A* and *B*). The CD21^low^CD27^−^ CD24^−^/CD11c^+^ phenotype of human ABCs is similar to ABCs found in mice^28^ (see Supplementary material online, *Figure S5C*). These CD21^low^CD27^−^CD24^−^ ABCs showed an even stronger association with age than the CD21^low^ B cells (*Figure 1G*).

### 3.2 ABCs are increased in CE cases and associated with incident CEs

**Figure 2:**
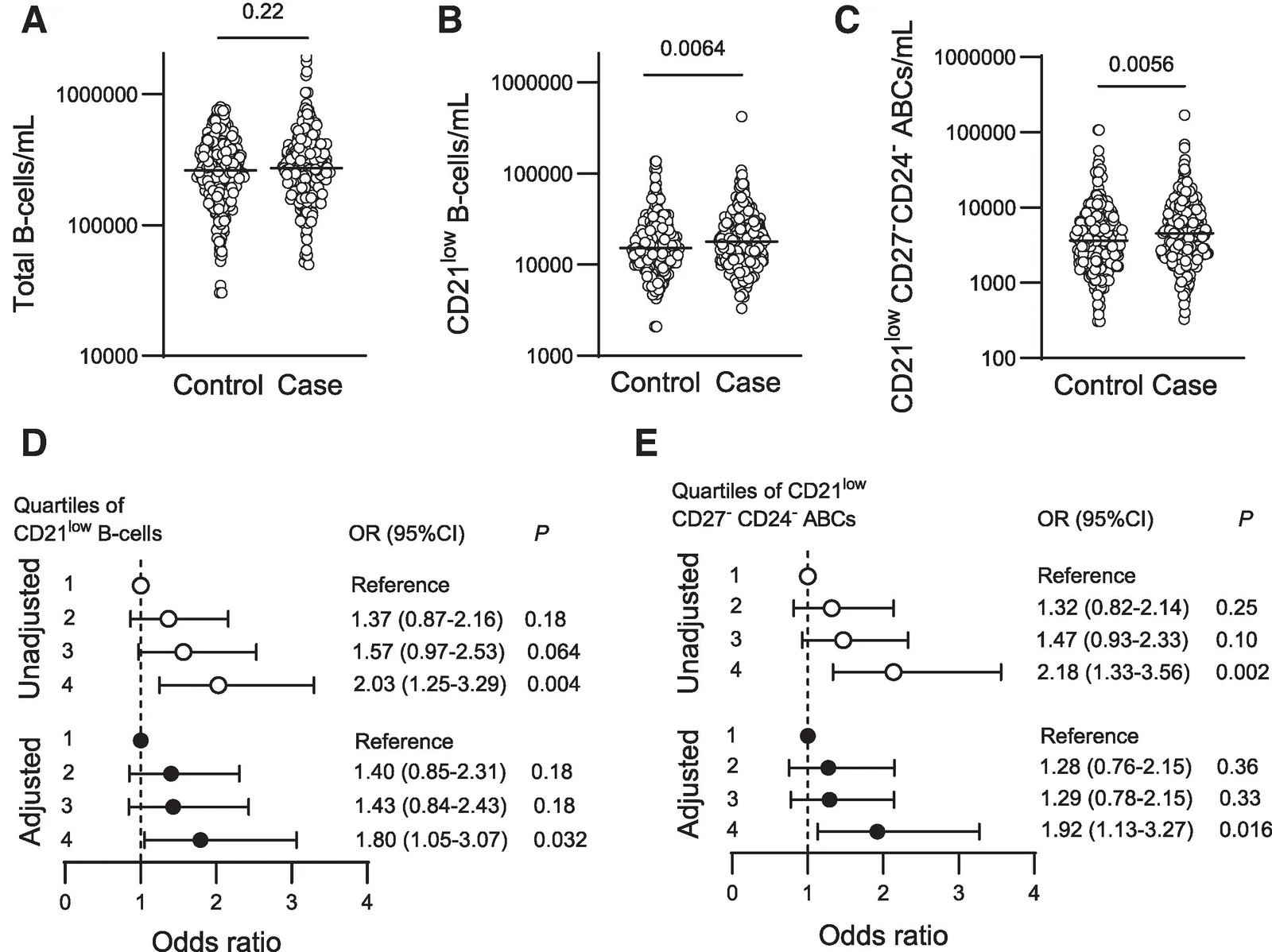
ABC counts are higher in CE cases vs. controls and associate with incident CEs. Cell counts per mL of blood of (*A*) total B cells, (*B*) CD21^low^ B cells, (*C*) CD21^low^CD27^−^CD24^−^ ABCs in CE cases (*N* = 302) and controls (*N* = 302). Data is presented as individual values and median and were analysed by Mann–Whitney tests. Associations between (*D*) quartiles of CD21^low^ B cells, (*E*) CD21^low^CD27^−^CD24^−^ ABCs and incident CE analysed by conditional logistic regression in an unadjusted model (302 case–control pairs) and a model adjusted for CVD risk factors (total cholesterol, HDL, systolic BP, diabetes, and current smoking; 279 case–control pairs). ORs with 95% CIs were compared with the lowest (reference) quartile.

Compared with controls, CE cases had higher counts of CD21^low^ B cells, CD21^low^CD27^−^CD24^−^ ABCs and IgM^−^ SM cells, while total B-cell counts were similar between the groups (*Figure 2C*, *Table 1*). Although several CD21^low^ B-cell subsets were found to be higher in cases compared with controls, the strongest statistical evidence for an increase in CE cases was found for CD21^low^CD27^−^CD24^−^ ABCs (*Table 1*, *Figure 2C*).

**Table 1:** Counts of B-cell populations in CE cases and controls

B-cell population (cells/mL blood)	Control (*N* = 302)	Coronary event case (*N* = 302)	*P*
Total B cells	2600 (177 300–382 900)	271 900 (193 600–390 700)	0.22
B2 cells	249 200 (172 000–369 600)	262 500 (186 500–372 100)	0.21
‘B1’ cells (CD27^+^ CD43^+^)	3400 (2200–5300)	3900 (2300–6100)	0.069
Plasmablasts (CD38^+^ CD24^−^)	1600 (900–2300)	1500 (900–2500)	0.76
Transitional B cells	13 000 (7500–19 800)	12 400 (7700–21 200)	0.65
Naïve B cells (IgD^+^ CD27^−^)	158 700 (99 800–244 800)	159 600 (107 100–258 600)	0.38
Switched memory (IgD^−^ CD27^+^)	32 600 (21 800–55 900)	41 500 (23 000–69 000)	**0**.**025**
IgM^−^ switched memory	26 500 (16 100–48 200)	32 700 (16 200–56 700)	**0**.**049**
IgM^+^ switched memory	5500 (3600–8600)	5500 (3100–9500)	0.87
Unswitched memory (IgD^+^ CD27^+^)	18 300 (11 300–27 200)	18 400 (11 800–28 000)	0.75
Double negative (IgD^−^ CD27^−^)	19 500 (13 500–30 900)	22 200 (14 800–32 700)	0.052
CD21^low^ B cells	15 100 (10 200–23 500)	17 800 (11 200–28 000)	**0**.**0064**
CD21^low^ naïve	4700 (3200–9000)	5800 (3400–10 400)	**0**.**013**
CD21^low^ naïve CD24^−^	1800 (1000–3300)	2200 (1100–4500)	**0**.**013**
CD21^low^ naïve CD24^+^	2900 (1700–4700)	3400 (2000–5600)	**0**.**015**
CD21^low^ double negative	3600 (2300–5600)	4100 (2600–6900)	**0**.**018**
CD21^low^ DN CD24^−^	1500 (900–2900)	2000 (1000–3500)	**0**.**018**
CD21^low^ DN CD24^+^	1700 (1100–2800)	1920 (1200–3000)	**0**.**011**
CD21^low^ switched memory	3500 (2100–5500)	3900 (2300–6700)	**0**.**021**
CD21^low^ unswitched memory	1800 (1000–3400)	1900 (1000–3400)	0.59
ABCs (CD21^low^ CD27^−^ CD24^−^)	3700 (2000–6600)	4600 (2400–8800)	**0**.**0056**

Statistical significances were analysed by Mann–Whitney tests. Statistically significant *P*-values between CE cases and controls are highlighted in bold.

Next, we applied conditional logistic regression to examine associations between B-cell populations and incident CE, accounting for relevant risk factors. We found that high counts (Quartile 4) of CD21^low^CD27^−^CD24^−^ ABCs were associated with incident CE with an odds ratio (OR) of 2.18 [95% confidence interval (CI) 1.33–3.56], *P* = 0.002, compared with low counts (Quartile 1) of CD21^low^CD27^−^CD24^−^ ABCs (*Figure 2E*). CE cases had higher levels of many CVD risk factors at study inclusion, including triglycerides, LDL, systolic BP, glucose, and HbA1c, and lower levels of HDL (*Table 2*). A regression model adjusted for CVD risk factors showed that CD21^low^CD27^−^CD24^−^ ABCs remained associated with incident CE independently of total cholesterol, HDL, systolic BP, diabetes, and current smoking with an OR of 1.92 (95% CI 1.13–3.27), *P* = 0.016, (*Figure 2E*). Additionally, adjustment for glucose and HbA1c did not affect the association, OR 1.94 (95% CI 1.14–3.31), *P* = 0.015. Likewise, CD21^low^CD27^−^CD24^−^ ABCs remained significantly associated with incident CE after additional adjustment for high-sensitivity C-reactive protein [OR 1.80 (95% CI 1.01–3.24), *P* = 0.049; Supplementary material online, *Table S6*], suggesting that the ABC-CE association is not simply driven by low grade inflammation. High counts of total CD21^low^ B cells were also associated with incident CE independently of total cholesterol, HDL, systolic BP, diabetes, and current smoking with an OR of 1.80 (95% CI 1.05–3.07), *P* = 0.032, (*Figure 2D*). CD21^low^ B cells encompass multiple subsets ascribed functionally distinct roles. Of note, several CD21^low^ B-cell subsets were significantly associated with incident CE (see Supplementary material online, *Table S7*). However, only CD11c-expressing CD21^low^CD27^−^CD24^−^ B cells remained independently associated with incident CE after adjustment for cardiovascular risk factors. In summary, ABCs were increased with age and independently predicted the risk of first-time CE in a general population-based cohort, even after adjusting for traditional risk factors.

**Table 2:** Baseline characteristics for CE cases and controls in the MDC study

	Control	Case	*P*	Total *N*
BMI (kg/m^2^)	25.6 (23.5–28.7)	26.0 (23.8–29.0)	0.29	602
Diabetes	41 (14%)	57 (19%)	0.08	604
Current smoker	67 (22%)	95 (31%)	0.01	604
Fasting glucose (mmol/L)	4.9 (4.6–5.3)	5.1 (4.7–5.6)	0.002	598
HbA1c (%)	4.8 (4.6–5.1)	5.0 (4.6–5.3)	0.001	592
Triglycerides (mmol/L)	1.2 (0.9–1.6)	1.4 (1.0–1.9)	0.002	595
Total cholesterol (mmol/L)	5.9 (5.3–6.7)	6.3 (5.5–7.1)	0.002	597
HDL (mmol/L)	1.3 (1.1–1.6)	1.2 (1.0–1.4)	<0.001	582
LDL (mmol/L)	4.1 (3.4–4.7)	4.4 (3.7–5.0)	0.002	577
eGFR (mL/min/1.73 m^2^)	76 (68–85)	75 (67–85)	0.53	586
BP-lowering treatment	53 (18%)	67 (22%)	0.15	604
Statin treatment	4 (1%)	9 (3%)	0.16	604
Diastolic BP (mmHg)	90 (80–96)	90 (82–96)	0.16	604
Systolic BP (mmHg)	142 (130–158)	148 (138–160)	0.004	604

Statistical significances analysed by Mann–Whitney tests for continuous variables and χ^2^ tests for categorical variables.

BMI, body-mass index; eGFR, estimated glomerular filtration rate; BP, blood pressure.

### 3.3 Women have higher counts of ABCs

Sex differences in immunity are well established, including that women have higher counts of B cells than men.^29^ We found that women had higher counts of almost all B-cell populations analysed, including CD21^low^ B cells and CD21^low^CD27^−^CD24^−^ ABCs (see Supplementary material online, *Figure S6A* and *Table S8*). Separate regression analyses for men and women showed that there was a CVD risk factor independent association between both CD21^low^ B cells and CD21^low^CD27^−^CD24^−^ ABCs, and incident CE in men, but not in women (see Supplementary material online, *Figure S6B* and *C*). Consequently, we concluded that while women have more ABCs, female sex is seemingly not driving the association between increased numbers of ABCs and incident CE.

### 3.4 ABCs accumulate in atherosclerotic mice upon aging

**Figure 3:**
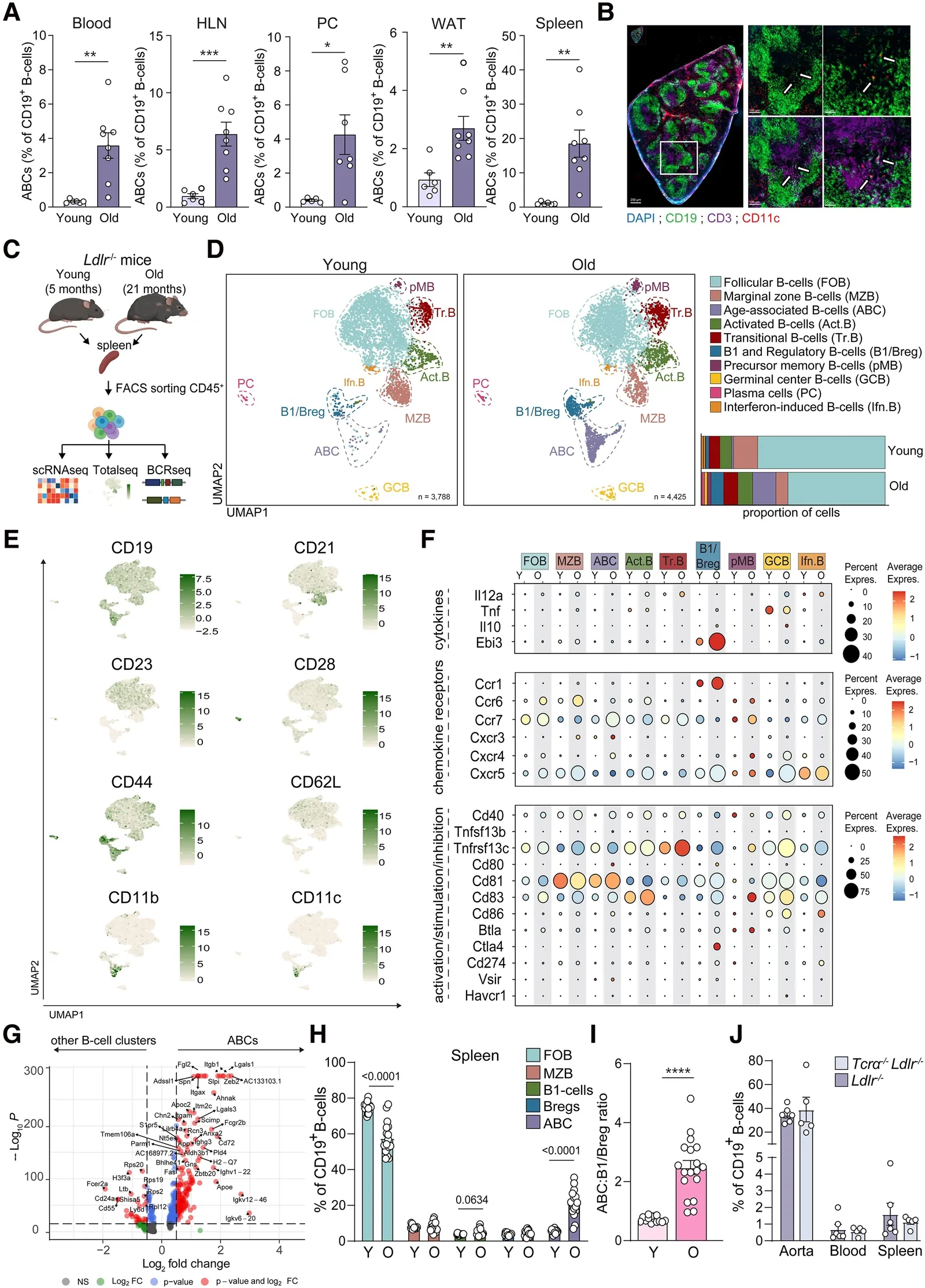
Single-cell profiling of splenic immune cells reveals accumulation of ABCs upon aging. (*A*) Frequencies of ABCs (% of CD19^+^ B cells) were measured with flow cytometry in blood, mediastinal lymph nodes (HLN), peritoneal cavity (Per), white adipose tissue (WAT) and spleen of young (5 months old) and aged (21 months old) female *Ldlr*^−/−^ mice. (*B*) Immunofluorescent staining (20×) of the spleen from an aged female *Ldlr^−/−^* mouse. CD19-FITC (green) was used to stain B cells, CD3-APC (purple) to stain T cells, CD11c-APC (red) to stain ABCs and DAPI (blue) to stain nuclei. (*C*) Workflow of single-cell RNA sequencing (scRNA-seq), Total-seq and BCR-seq on splenic CD45^+^ cells of CD-fed 5 months old (pooled from *n* = 7) and 21 months old (pooled from *n* = 8) female *Ldlr*^−/−^ mice. (*D*) Uniform Manifold Approximation and Projection (UMAP) visualization (square panels) and stacked proportional diagram of clustered splenic B cells. (*E*) Surface protein (Total-seq) expression of B-cell markers projection on the UMAP. (*F*) Average gene expression of cytokines, chemokine receptors, and activation-, stimulatory-, and inhibitory molecules. (*G*) Volcano plot displaying differential gene expression between the ABC cluster and other B-cell clusters. (*H*) Percentages of FOBs, MZBs, B1 cells, Bregs and ABCs in the splenic CD19^+^ B-cell compartment of young (Y) and old (O) *Ldlr*^−/−^ mice were measured with flow cytometry. (*I*) In these young and old mice, the ratio between ABC and atheroprotective B1 + Breg percentages was calculated. (*J*) ABC percentages in the aorta, blood and spleen of *Tcrα^−/−^ Ldlr^−/−^* mice (44–51 weeks) were compared with those in *Ldlr^−/−^* mice (52–53 weeks). Data are from *n* = 5–8 mice per group. Statistical significance was tested by one-way ANOVA. Mean ± SEM plotted. **P < 0.01, ***P < 0.01, ****P < 0.0001. Graphical elements were created with Biorender.com.

The observed association between ABCs and incident CE in humans does not establish a causal link between increased ABCs and CVD. To be able to explore the direct contributions of ABCs to atherosclerosis development we turned to study experimental atherosclerosis and changes in B-cell immunity in young (5 months) and aged (21 months) female *Ldlr^−/−^* mice fed a CD. While absent in young mice, ABCs (defined as CD19^+^CD23^−^CD21^low^CD11b^+^/CD11c^+^) constituted on average 3–20% of the CD19^+^ B-cell compartment in the circulation and several tissues of aged mice and were particularly abundant in the spleen (*Figure 3A*). We further confirmed the presence of ABCs in the spleen by immunofluorescent staining, indicating that ABCs are located at the T-B border zones (*Figure 3B*; white arrows, orange signal). As the spleen is important for the orchestration of atherosclerosis-specific systemic adaptive immunity, we mapped the transcriptome of splenic CD45^+^-cells isolated from young and aged female *Ldlr^−/−^* mice using 5′ single-cell RNA-sequencing (scRNA-seq) paired with V(D)J/B-cell receptor (BCR)-seq and surface protein profiling via Total-seq (*Figure 3C*, Supplementary material online, *Figure S3*). Unsupervised clustering resulted in clusters containing mainly B cells and T cells, and to a lesser extent natural killer (NK)-cells and myeloid cells (see Supplementary material online, *Figure S7* and *Table S9*). We continued with *Cd79a*^+^ and *Cd19*^+^ B-cell clusters to gain more granular insights into B-cell immunity. B-cell subclustering resulted in 10 clusters (*Figure 3D*, Supplementary material online, *Figure S7B* and *C* and *Table S10*) identified as FOB (*Fcer2a*), marginal zone B cells (MZB; *Cr2*, *Cd1d1*), activated B cells (Act.B; *Cd83*, *Myc*), transitional B cells (Trans.B; *Cd24a*, *Cd93*), memory B cells (pMB; *Bach2*, *Cd22*), germinal centre B cells (GCB; *Fas*, *Aida*, *Mki67*, *Mef2b*), plasma cells (PCs; *Xbp1*, *Sdc1*, *Prdm1*), interferon-induced B cells (Ifn.B; *Ifit3*, *Usp18*, *Irf7*), ABCs (*Itgam*, *Itgax*, *Tbx21*), B1 (*Zbtb32*) and regulatory B cells (B1/Breg; *Cd9*, *S100a6*). Notably, the proportions of FOBs and MZBs were reduced in aged compared with young mice, whereas ABCs and B1/Bregs were substantially expanded (*Figure 3D*).

To confirm the subclustering of B cells, we analysed the labelling of surface proteins with feature barcoding (Total-seq). In line with scRNA-seq data, we found high expression of CD23 on FOBs and CD21 on MZBs (*Figure 3E*, Supplementary material online, *Figure S7B*). CD28 was found on PCs and ABCs, and B1/Bregs were CD44^hi^ (*Figure 3E*). Protein expression of CD11b, CD11c and low levels of CD21 and CD23 on the ABC cluster were also confirmed with Total-seq (*Figure 3E*).

The expression signatures of the identified B-cell populations were in line with functional characteristics of different B cells. The regulatory signature of Bregs was confirmed by expression of *Il10* and the IL-35 subunit *Ebi3* (*Figure 3F*). Notably, expression of *Ebi3* was markedly higher in aged compared with young mice (*Figure 3F*). Although gene expression of cytokines was comparably low in ABCs, a portion of these cells expressed high levels of genes encoding co-stimulatory and co-inhibitory molecules *Cd80*, *Havcr1* (Tim-1), and *Vsir* (VISTA; *Figure 3F*). Furthermore, ABCs of aged mice expressed chemokine receptor gene *Cxcr3* that is associated with PC differentiation and migration towards sites of inflammation in autoimmune diseases, including multiple sclerosis and axial spondyloarthritis.^30–32^ In addition, other genes associated with PC differentiation and survival (*Zbtb20*, *Lgals1*, *Lgals3*, *Itm2c*) were differentially expressed in ABCs, including the transcription factor *Zeb2*, which has recently been identified as a key driver of ABC formation^33^ (*Figure 3G*). Taken together, the scRNA-seq data in young and aged mice identified distinct B-cell populations with gene expression patterns consistent with functional specialization and indicated changes with aging.

Next, we used flow cytometry to confirm that the proportion of splenic FOBs and MZBs decreased upon aging, while ABCs expanded to become the second largest B-cell population in aged mice (*P* < 0.01; *Figure 3H*, Supplementary material online, *Figure S8*). Although the frequency of atheroprotective B1 cells also slightly increased, aging resulted in an elevated pro-inflammatory ABC to B1/Breg ratio (*Figure 3I*). In addition, we observed that ABCs were present in TCRα-deficient mice (44–51 weeks) to a similar extent as in TCRα-sufficient controls (52–53 weeks) in aorta, blood and spleen (*Figure 3J*), indicating that their development can occur independently of T cells. Together these results show that ABCs are the main B-cell population that expands with age both in a human population and in laboratory mice and that this process is intrinsic to B cells rather than dependent on T-cell help.

### 3.5 Adoptive transfer of ABCs aggravates atherosclerosis

**Figure 4:**
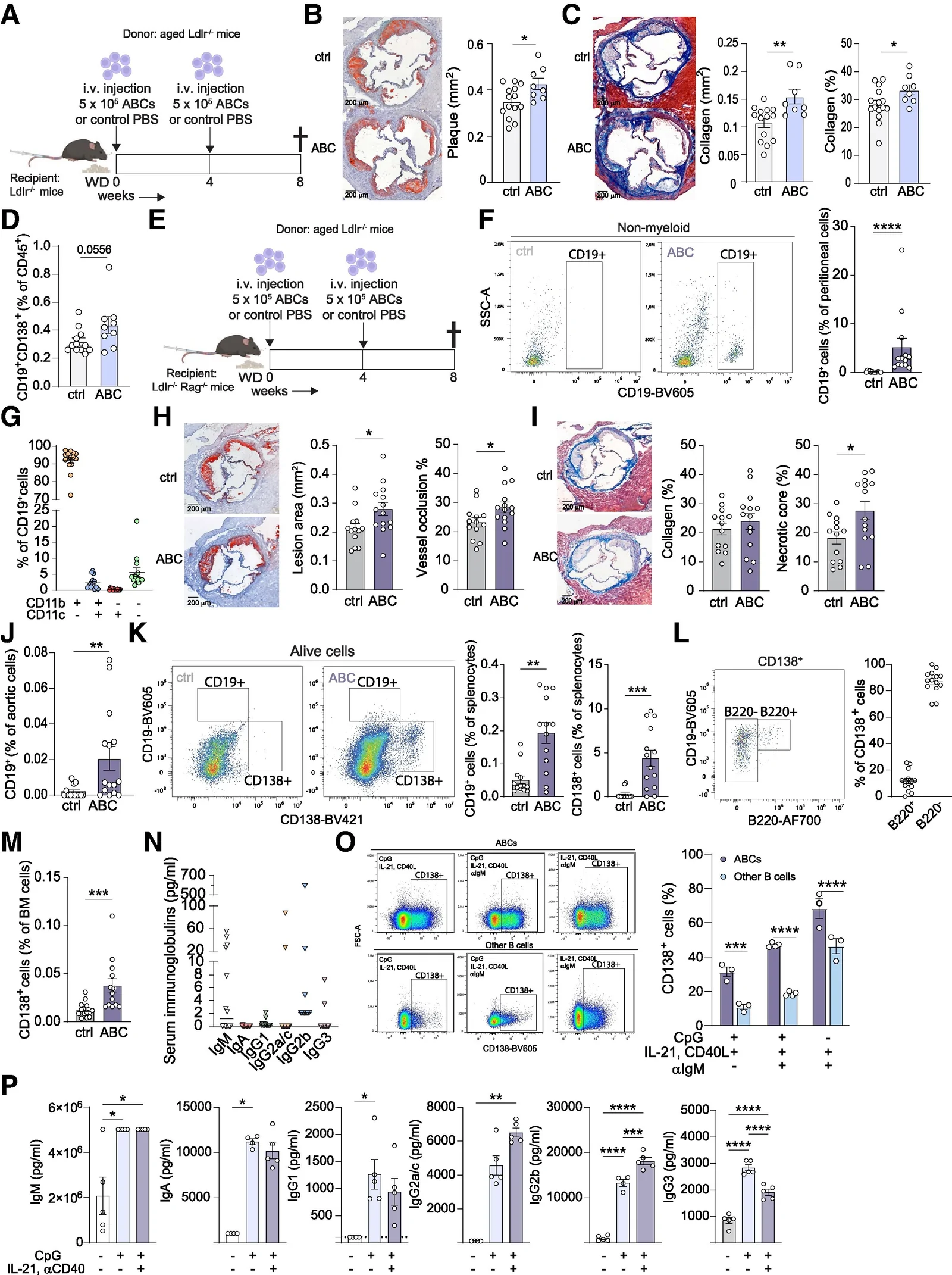
Transfer of ABCs promotes atherosclerosis in immunodeficient mice. (*A*) Experimental set-up: ABCs were isolated from aged female *Ldlr*^−/−^ mice (18–21 months old) and transferred at *T* = 0 weeks and at *T* = 4 weeks, vs. control vehicle to *Ldlr^−/−^* mice (*n* = 8/group) and put on a WD for 8 weeks, starting at *T* = 0. After 8 weeks of WD (*B* and *C*) cross sections of the aortic root were stained with Oil-Red-O and Masson’s trichrome. (*B*) Lesion size and (*C*) collagen content were analysed in ABC recipient (light purple) and control (light grey) *Ldlr^−/−^* mice. (*D*) The percentage of splenic CD19^+^ CD138^+^ plasmablasts was measured using flow cytometry. (*E*) Experimental set-up: ABCs were isolated from aged female *Ldlr*^−/−^ mice (18–21 months old) and transferred (*n* = 14) at *T* = 0 weeks and at *T* = 4 weeks, vs. control vehicle (*n* = 13) to *Ldlr^−/−^Rag1^−/−^* mice and put on a WD for 8 weeks, starting at *T* = 0. After 8 weeks of WD (*F*) percentages of CD19^+^ B cells in the peritoneal cavity were measured. (*G*) Proportions of CD11b^+^CD11c^−^, CD11b^+^CD11c^+^, CD11b^−^CD11c^+^, and CD11b^−^CD11c^−^ cells within peritoneal CD19^+^ B cells of ABC recipients. (*H* and *I*) Cross sections of the aortic root were stained with Oil-Red-O and Masson’s trichrome. (*H*) Lesion size and vessel occlusion were quantified. (*I*) Collagen content and necrotic core were measured as percentage of lesion area. (*J*) Percentage CD19^+^ B cells of aortic cells after transfer. (*K*) Percentages of CD19^+^ B cells and CD138^+^ cells in the spleen of *Ldlr^−/−^Rag1^−/−^* mice were quantified. (*L*) Frequencies of B220^−^ and B220^+^ cells within CD138^+^ cells were measured in spleens of ABC-recipient mice. (*M*) CD138^+^ cells were measured in the BM of *Ldlr^−/−^Rag1^−/−^* mice. (*N*) Immunoglobulin levels were quantified in serum of ABC recipients using LEGENDplex. (*O*) *In vitro* PC differentiation within the ABC population and other B cells after 6 day stimulation of isolated B cells with IL-21, CD40L, CpG and/or anti-IgM (*n* = 3–4). (*P*) Measurement of immunoglobulins secreted by ABCs (*n* = 5) stimulated with TLR9-ligand CpG in a T-cell-independent and T-cell-dependent (IL-21, anti-CD40) setting. Statistical significance of two groups was tested by two-tailed *t*-test or Mann–Whitney test. Statistical significance of three groups was tested by one-way ANOVA or Kruskal–Wallis test. Mean ± SEM plotted. All replicates represent biological replicates. *P < 0.05, **P < 0.01, ***P < 0.001, ****P < 0.0001. Graphical elements were created with Biorender.com.

To determine if ABCs can contribute to atherosclerotic plaque development in mice, we sorted alive CD19^+^CD23^−^ B cells expressing CD11b/CD11c from aged female *Ldlr*^−/−^ (21 months old) mice and adoptively transferred ABCs or control vehicle into young female *Ldlr^−/−^* mice (2–3 months old), which lack ABCs (*Figure 4A*, Supplementary material online, *Figure S9A*). After 8 weeks of WD, cholesterol levels were similar between groups (see Supplementary material online, *Figure S9B*). ABC-recipients developed increased atherosclerotic lesion burden (*Figure 4B*), with enhanced collagen content (*Figure 4C*), demonstrating the pro-atherogenic potential of ABCs. These effects were comparable to that induced by FOBs, a well-established pro-atherogenic B-cell subset (see Supplementary material online, *Figure S9C*). In parallel, we observed a trend towards more plasmablasts in the spleen of ABC-recipients (*Figure 4D*). Because young *Ldlr^−/−^* mice possess a complete immune system, including endogenous B cells, we also transferred ABCs to female *Ldlr^−/−^Rag1^−/−^ mice* (2–3 months old) to gain more mechanistic insights. At termination, we retrieved CD19^+^ B cells from the peritoneum of ABC-recipient mice, which retained their ABC phenotype (*Figure 4E* and *F*, Supplementary material online, *Figure S9D*). Total serum cholesterol levels did not differ between ABC-recipient and control mice (see Supplementary material online, *Figure S9E*). Analysis of plaque development in the aortic root showed that ABC transfer significantly increased atherosclerosis compared with controls (ABC: 0.28 ± 0.02 mm^2^ vs. ctrl: 0.21 ± 0.02 mm^2^, *P* < 0.05, *Figure 4G*), confirming that ABCs promote plaque progression. Although collagen content (*Figure 4H*) was similar, plaques from ABC-recipients showed increased necrotic core formation (*Figure 4I*). In addition, we observed an increase in the frequency of CD19^+^ B cells in the aorta of ABC-recipients (*Figure 4J*), while other aortic and splenic leukocyte numbers and frequencies of CD11b^+^ myeloid cells, neutrophils and CD161^+^ NK-cells were comparable between the groups, as were serum cytokine levels (see Supplementary material online, *Figure S9F*–*H*). In conclusion, adoptive transfer of ABCs promoted plaque progression.

### 3.6 ABCs differentiate into PCs and home to spleen and BM

Next, we explored the phenotype of the B cells recovered from ABC-recipient mice in more depth. Given our observation of increased expression of genes associated with PC differentiation in ABCs from aged mice, we wondered whether ABCs had a predisposition to differentiate into PCs. Eight weeks after the transfer of ABCs to *Ldlr^−/−^Rag1^−/−^* mice we found that <0.5% of the splenocytes were CD19^+^ B cells (*Figure 4K*). Most of the transferred CD19^+^ ABCs had differentiated into CD138^+^ cells in the spleen, the majority being CD19^−^CD138^+^ and B220^−^CD138^+^ PCs (*Figure 4K* and *L*). Furthermore, we detected CD138^+^ cells in the BM of ABC recipients (*Figure 4M*). We next analysed the serum of ABC-recipient mice for presence of antibodies. Although antibody levels were below detection limit in some ABC-recipient mice, we observed low serum titres of IgM, IgG1, IgG2a/c, IgG2b, and IgG3 (*Figure 4N*). Culture of B cells, isolated from aged atherosclerotic mice, in the presence of CD40L, IL-21 and/or CpG and anti-IgM, confirmed that ABCs can differentiate into CD138^+^ PCs (*Figure 4O*). Notably, this differentiation was more pronounced within ABCs compared with CD19^+^CD11b^−^CD11c^−^ B cells. ABC-derived PCs, generated by stimulation of ABCs isolated from aged mice with the TLR9 ligand CpG (T-cell-independent) in the presence or absence of IL-21 and anti-CD40 (T-cell-dependent), secreted a diverse repertoire of antibodies, regardless of whether the stimulation was T-cell-dependent or -independent (*Figure 4P*), to a higher extent than FOBs (see Supplementary material online, *Figure S9*).

### 3.7 Single-cell BCR profiling shows clonal expansion of ABCs

**Figure 5:**
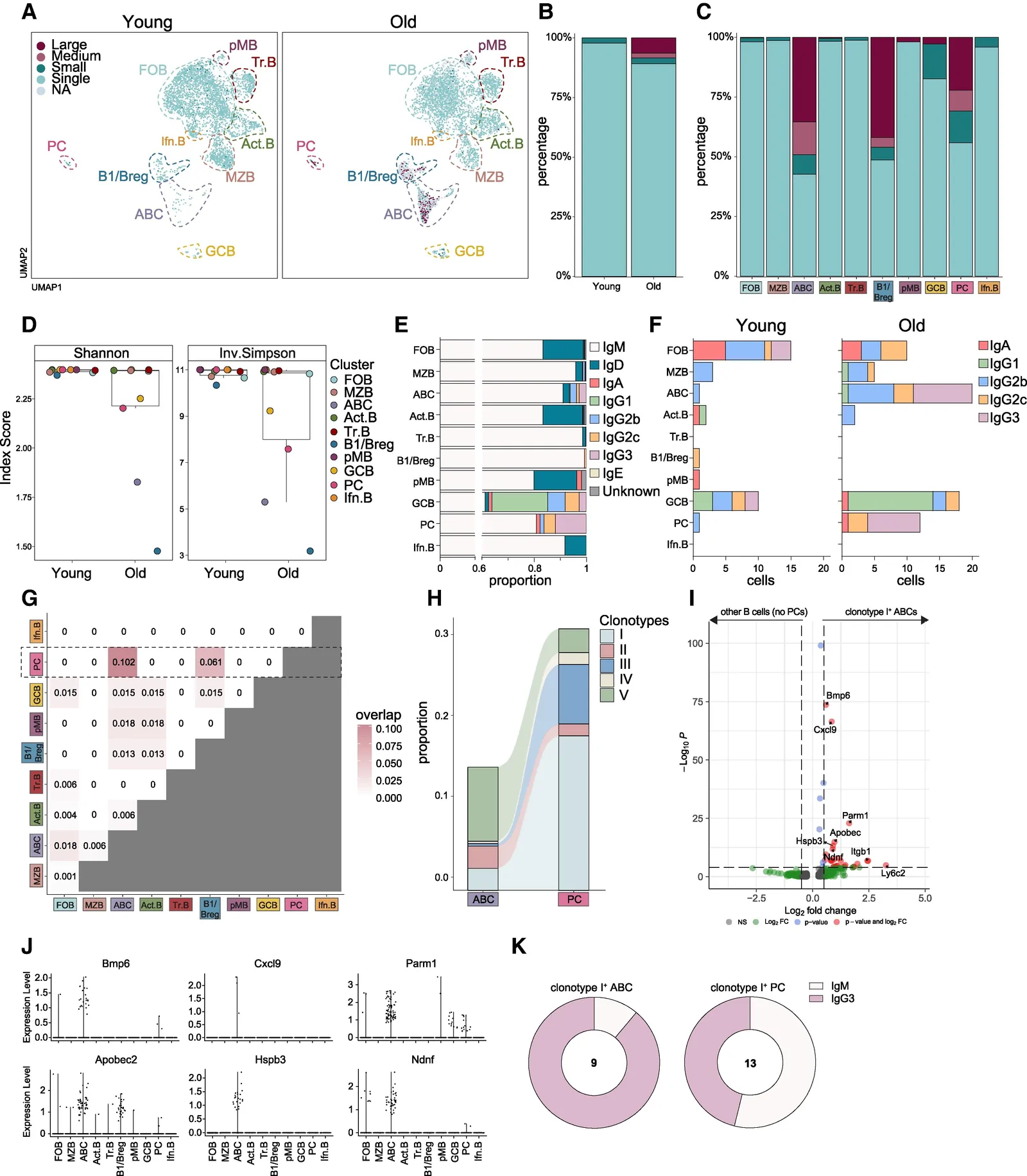
ABCs clonally expand and show clonal overlap with PC. (*A*) UMAP projection of clonally expanded B cells in the spleen, where BCR clonotypes are categorized based on the frequency in the single clonal expansion category (0 < *X* ≤ 1; light teal), small clonal expansion category (1 < *X* ≤ 5; dark teal), medium clonal expansion category (5 < *X* ≤ 20; light red), large clonal expansion category (20 < *X* ≤ 100; dark red) and B cells of which no clonotypic information is known (NA; grey). The percentage of single cells in each clonal expansion category (*B*) per age group, and (*C*) per cluster is shown. (*D*) Clonal diversity of each cluster per age group as measured by Shannon and Inverse Simpson diversity indices. (*E*) The proportion of IgM, IgD, IgA, IgG1, IgG2b, IgG2c, IgG3, and IgE expressing cells in each cluster. (*F*) The number of isotype-switched cells expressing IgA, IgG1, IgG2b, IgG2c, IgG3. (*G*) The proportion of clonal overlap between clusters. (*H*) Frequency of clonal overlap between ABCs and PCs. (*I*) Volcano plot showing differential gene expression between ABCs expressing clonotype I and other B cells excluded from PCs, of which expression of selected differentially expressed genes is displayed in (*J*) violin plots. (*K*) Isotype distribution of clonotype I-expressing ABCs and PCs, with the number of total cells expressing clonotype I shown in the centre of the circle. Data are from CD-fed 5 months old (pooled from *n* = 7) and 21 months old (pooled from *n* = 8) female *Ldlr*^−/−^ mice.

Upon antigen encounter, activation, and clonal expansion, B cells can differentiate to PCs. We performed V(D)J sequencing analysis to profile the BCR repertoire and characterize the clonal composition of splenic B cells in *Ldlr^−/−^* mice (*Figure 3B*). Clonal expansion in B cells from CD-fed young mice was minimal as clonotypes were expressed by either a single B-cell (Single) or by 1–5 B cells (Small; *Figure 5A* and *B*). In contrast, aging induced a large clonal expansion of ABCs and B1/Bregs in CD-fed *Ldlr^−/−^* mice, as most of these cells contained a clonotype that was present on 5–20 other B cells (Medium) or on 20–100 other B cells (Large). Clonal analysis of individual clusters indeed showed that clonally expanded B cells were primarily present in ABCs, B1/Bregs, GCB and PCs (*Figure 5C*). In line with this data, clonal diversity was reduced in aged mice as indicated by the lower Shannon entropy and Inverse Simpson index (*Figure 5D*).

Next, we investigated the isotype distribution of detected clonotype-expressing cells in each cluster (*Figure 4E*). Notably, IgM was the most abundant isotype in all clusters. In *Ldlr^−/−^* mice, GCBs contained the highest percentage of isotype-switched cells compared with other clusters, as seen by the proportion of IgG1, IgG2b, IgG2c, and IgG3-expressing GCBs. When stratified by age, class-switched cells were most abundant in the FOB cluster in young mice, while ABCs held the largest number of class-switched B cells expressing IgG2b, IgG2c, and IgG3 isotypes in aged mice (*Figure 5F*). Like ABCs, relatively many PCs expressed the IgG3 isotype. To determine clonotypic similarity between different B-cell types, we measured the proportion of overlapping clones with the Morisita–Horn index, which assigns 0 and 1 values to unrelated and identical clonotypes,^34^ respectively (*Figure 5G*). Interestingly, we found the highest clonal overlap between ABC and PCs, as 5 of the 49 unique clonotypes found in the PC cluster were present in the ABC cluster (*Figure 5H*). Clonotype III was also expressed by the B1/Breg cluster, while the other four clonotypes were exclusively expressed by ABCs and PCs. These five clonotypes were present on 30% of all PCs, indicative of their large clonal expansion within the PC cluster. Together, these findings can be explained by differentiation of ABC clonotypes into antibody-producing PCs. To further examine clonotype I-expressing ABCs, which was the largest expanded ABC-specific clonotype in the PC cluster, we investigated the differential gene expression of these cells in comparison to all other B cells (except PCs) to detect evidence of PC differentiation. Indeed, we observed that PC differentiation genes *Ly6c2* and *Itgb1* were differentially expressed by clonotype I^+^ ABCs (*Figure 5I*). Interestingly, clonotype I^+^ ABCs displayed differential expression of *Bmp6*, *Cxcl9*, *Parm1*, *Apobec2*, *Hspb3*, and *Ndnf*, which seemed to be expressed almost exclusively by ABCs (*Figure 5J*).

## 4. Discussion

Aging may be the most important risk factor for developing CVD. In this study, we demonstrate that aging is associated with increased circulating ABCs in humans (especially above 65 years of age) and in old atherosclerotic mice. We show that a high frequency of ABCs is associated with incident CE independently of CVD risk factors, suggesting that ABCs alone contribute to CVD prediction without support or interaction with traditional risk factors. Our preclinical data show a pro-atherogenic role for ABCs, supporting an emerging concept where ABCs accumulate through clonal expansion during aging and possibly contribute to atherosclerosis development and CVD by being a precursor or source for PC differentiation. Such clonal expansion and accumulation of ABCs could potentially explain the strong association between aging and CVD.

Human ABCs have been referred to as CD21^low^ B cells, IgD^−^CD27^−^ (DN) B cells or atypical B cells, all describing broadly overlapping B-cell populations. Within this landscape, more refined subpopulations of CD21^low^ B cells have recently been defined based on IgD, CD27, and CD11c expression, such as DN2 (CD21^low^CD11c^+^), DN3 (CD21^low^CD11c^−^), IgD^−^CD27^low^, CD11c^+^ UM (IgD^+^CD27^+^IgM^−^) and activated naïve B cells (IgD^+^, CD27^−^CD11c^+^).^22,35^ Notably, the human CD11c-expressing ABC populations (DN2 and activated naïve), have been described to also lack CD24 expression.^36^ These phenotypic features align with our definition of ABCs (CD21^low^CD27^−^CD24^−^), in which loss of CD24 coincided with CD11c expression. Similarly, the ABC population in mice has been described to be dynamic and heterogeneous, with diverse expression of CD11c, CD11b, and T-bet.^24,37,38^ While ABCs were absent in young *Ldlr^−/−^* mice, ABCs proportionally became the second largest B-cell population in spleens of aged *Ldlr^−/−^* mice at the cost of FOBs and MZBs. Although our human study cohort included middle-aged or older individuals, we also observed a significant increase in the number of total ABCs with age. Recently, Pattarabanjird *et al*.^22^ found that total CD11c^+^ and the DN2 B cells were associated with age and high vs. low extent of atherosclerotic disease quantified by coronary angiography in cardiac catheterization patients (*n* = 60). Here we extend these findings to a much larger cohort without clinically manifested disease at baseline and with a long follow up for CE. We found that total CD21^low^ B cells, and in particular CD21^low^CD27^−^CD24^−^ ABCs were associated with incident first-time CE independently of CVD risk factors. The functional impact of ABC accumulation is likely to depend not only on their absolute numbers, but also on their balance relative to other cells such as regulatory B cells. Thus, the potentially pathogenic role of ABCs needs to be understood in the light of their interactions and balance with other immune cells. In our murine analyses, aging was accompanied by a disproportionate increase in ABCs compared with atheroprotective B1/Breg cells, resulting in an elevated ABC:B1/Breg ratio. These findings suggest that aging leads to an imbalance within the B-cell compartment, shifting towards a more pro-inflammatory state. In this context, the potentially pathogenic role of ABCs may reflect both their numerical expansion and a concurrent relative reduction in regulatory B-cell populations, together contributing to immune dysregulation and increased cardiovascular risk with aging.

Using single-cell transcriptomic analysis, we report that ABCs in the spleen of aged *Ldlr^−/−^* mice show increased expression of effector molecules *Cd80* and *Hvcar1*, similar to ABCs isolated from the aortas of aged *Ldlr^−/−^* mice.^21^ Combination of single-cell transcriptomic data with paired clonal analysis revealed that ABCs clonally expand with a high extent of class-switching and share clonotypes with PCs. These observations indicate that ABCs are directly related to and can differentiate into antibody-secreting PCs in atherosclerotic mice. Mechanistically, we provide evidence for this concept as transferred ABCs in *Ldlr^−/−^Rag1^−/−^*recipient mice differentiated into CD138^+^ PCs in the spleen and also homed to the BM. Consistent with the known heterogeneity of ABCs,^24^ where CD11c^+^ ABCs are particularly prone to differentiate into antibody-secreting PCs,^39,40^ the retrieved ABC population was predominantly of the CD11b^+^CD11c^−^ phenotype, likely reflecting both PC differentiation of the CD11c^+^ subset and CD11c downregulation during expansion and effector transition. Moreover, we detected IgM, IgG1, IgG2c, IgG2b, and IgG3 antibody secretion by ABCs from aged atherosclerotic mice *in vitro* but also *in vivo*, in serum of ABC-recipient *Ldlr^−/−^Rag1^−/−^* mice. These observations are in agreement with previous work, where it has been elegantly shown that transferred ABCs gave rise to CD138^+^ PCs within 7 days after transfer, which were still present at 21 days.^24,41^ Similarly, transcriptomic research in mice has indicated that ABCs are poised for PC differentiation and antibody secretion. Next, we showed that adoptive transfer of ABCs from aged mice to young *Ldlr^−/^-^−^* and *Ldlr^−/−^Rag1^−/−^-*recipients aggravated atherosclerosis development, indicating that ABCs inherently promote atherosclerosis. In addition, plaques of ABC-recipient mice displayed features of more advanced atherosclerosis, including collagen deposition and enlarged necrotic core areas, supporting an overall pro-atherogenic role of ABCs, both in the absence and presence of competing or interacting T and B cells. In support of these findings, we and others have shown that ABCs can become activated and differentiate into antibody-secreting PC independent of T-cell help, via TLR signalling, cytokine exposure and/or BCR stimulation.^24,37,38,42–45^ In line, we found that ABCs developed in TCRα-deficient mice at similar levels as in controls, supporting that their emergence does not require T cells. Moreover, the observed pro-atherogenic role of ABCs is in line with a recent study, that showed reduced atherosclerosis in 6 months old *Apoe*^−/−^ mice with a B-cell-specific T-bet deletion,^23^ although these mice do not fully display an aged phenotype. Previous studies on antibody deficiency in atherosclerosis indicate that the net impact of humoral immunity is context-dependent, with Prdm1-dependent loss of antibodies attenuating atherosclerosis,^46^ whereas Xbp1 deficiency aggravates lesion development and necrotic core formation,^47^ suggesting that distinct antibody specificities and isotypes can have opposing effects on plaque biology. In our setting, the overall pro-atherogenic effect of ABC transfer suggests that any potentially protective antibodies are outweighed by pathogenic activities of ABC-derived antibodies or other effector functions, underscoring the need for future work dissecting the antigen specificity and isotype profile of ABC-derived immunoglobulins in atherosclerosis. Based on prior work on atherosclerosis-associated autoantigens, plausible ABC targets include oxidation-specific epitopes (such as oxidized LDL and its modified apolipoprotein B-100 neoepitopes), apoptotic cell–derived antigens, and post-translationally modified self-proteins within the inflamed arterial wall, all of which may fuel chronic vascular inflammation and lesion progression when targeted by class-switched antibodies.^48^ Beyond antibody production, ABCs can act as potent antigen-presenting cells^20^ and may interact with T cells and other immune subsets within plaques or arterial tertiary lymphoid structures, while their association with pro-inflammatory cytokines such as IFNγ, TNF, IL-6 and IL-21 in other chronic inflammatory diseases supports a model in which ABC-derived cytokines amplify vascular inflammation and plaque growth.^49^ Future studies are needed to define the localization of ABCs and their progeny in the atherosclerotic microenvironment. In the current study, precise spatial localization of ABCs in murine plaques has proven challenging due to plaque autofluorescence and the low frequency of ABCs, which may be underrepresented in histological sections. Alternative approaches such as spatial transcriptomics may be needed to provide further insight into how ABCs react to antigens and interact with local immune cell networks to drive disease progression.

There are some limitations in the current study that need to be acknowledged. First, our study does not pinpoint the exact mechanism by which ABCs contribute to atherosclerosis and CE. Although data in our clinical cohort indicates that ABCs can predict future CE on a population basis, counts of ABCs cannot be used for prognosis on an individual level and causality cannot be inferred. Our data in mice suggest that the differentiation of PCs from ABCs is a plausible mechanism, but this must be formally shown in future studies. Second, the scarcity of ABCs restricts the study of specific ABC subpopulations with sufficient statistical power, and our case-control matching (one control per case) may have reduced the ability to detect weaker associations with cardiovascular risk. Third, the current study only examines associations between ABCs and first-time CE, and future studies should also assess associations with atherosclerotic burden, secondary events and disease severity to understand whether ABCs contribute to atherogenesis or the transition to plaque rupture. Additionally, the current study cohort was recruited over 30 years ago and it is becoming increasingly evident that changes in risk factor exposures and management has changed how CVD manifests.^50^ Thus, our findings must be verified in more contemporary populations. Moreover, a better understanding of the functional role of the different ABC subpopulations will likely be needed to enable studies with even better power. Understanding their role in both health and disease, both in CVD and other autoimmune conditions, will also be important as it is a promising prospect to target or delete ABCs to cure many ailments, particularly if no health promoting function is compromised.

In conclusion, our findings show that ABCs that clonally expand and accumulate with increasing age, can predict incident CE and can cause atherosclerosis development when transferred from aged to young mice. Interestingly, ABCs emerge as potential therapeutic targets that, if deleted or stopped from accumulating, may slow atherosclerosis development and prevent clinical events in our aging population.

Translational perspective Aging drastically increases the risk for acute cardiovascular events caused by atherosclerosis. This study shows a significant and promoting role for ABCs in the immunopathology of atherosclerosis. ABCs accumulate during aging in atherosclerotic mice and humans. In humans, ABCs are associated with incident first-time CEs. In mice, we show that ABCs are highly clonal, differentiate into PCs and contribute to atherosclerosis development. From a clinical perspective, these findings suggest that circulating ABCs hold promise as a risk predictor and therapeutic target to halt atherosclerosis progression and CEs.

## Supplementary Material

cvag154_Supplementary_Data

## Data Availability

*In silico* data analysis was performed using custom R scripts (R version 4.2.2) designed especially for this research and/or based on the recommended pipelines from the pre-existing packages listed in the individual segments above. Single-cell RNA sequencing data are available upon reasonable request to A.C.F. The data that support the findings of the human study are available from the authors (P.K. or H.B.) upon reasonable request, but, due to some of the data containing information that could compromise research participant privacy or consent, requests may require ethical review and full compliance to the general data protection regulation.
