# Supplementary material for: Age-associated B cells predict coronary events in humans and aggravate murine atherosclerosis
Source: Cardiovasc Res. 2026 Jul 13;122(13):1789–802. doi: 10.1093/cvr/cvag154 (PMC13604844; doi:10.1093/cvr/cvag154)
Supplement: Supplementary file 1 [file cvag154_supplementary_data.zip › 15072026_SuppFigures.pdf]

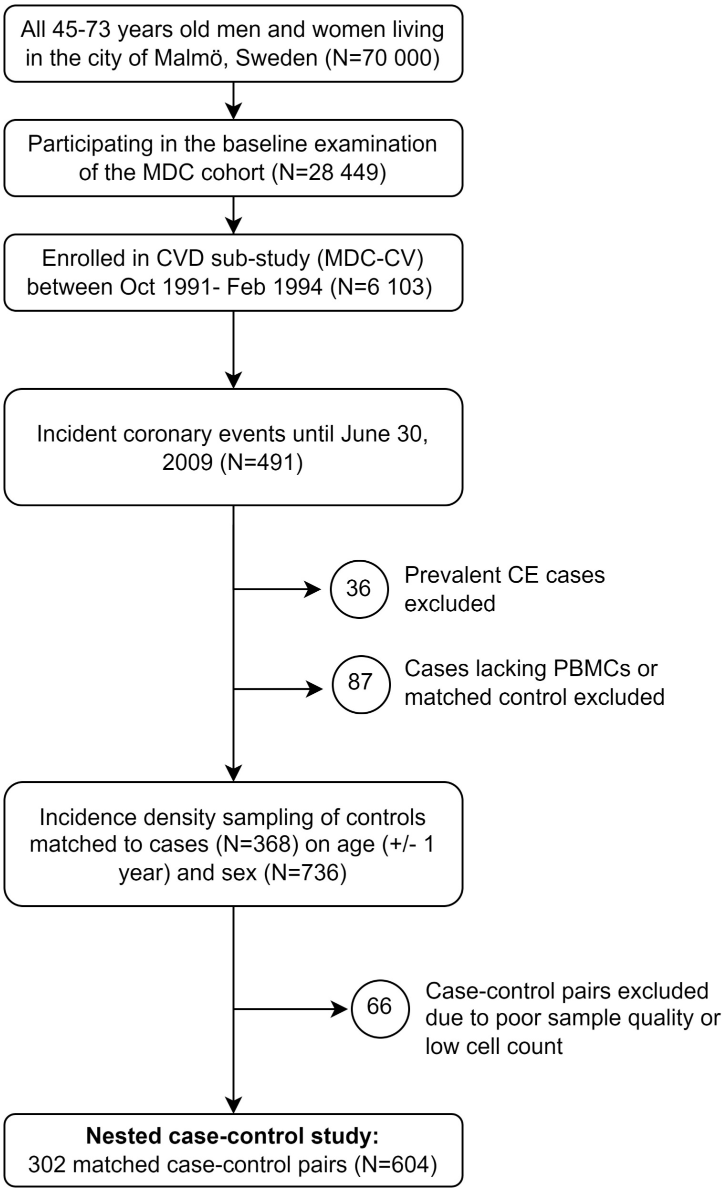

Supplemental Figure 1. Study design of the case-control study that was nested within the population-based Malmö Diet and Cancer (MDC) study. During the follow-up period, 491 incident coronary event (CE) cases, corresponding to 8.0% of the CVD-sub study (MDC-CV) participants, were retrieved by data linkage to the Swedish Hospital Discharge Register and the Cause of Death Registry of Sweden. Prevalent CE before study inclusion and participants who lacked a peripheral blood mononuclear cell (PBMC) sample or could not be matched to a control were excluded. Remaining participants with an incident CE were matched to a control using incidence density sampling based on age (±1 year) and sex. After flow cytometric analysis, some case-control pairs had to be excluded due to poor sample quality after thawing, low cell count, or non-adherence to the standard operating procedures, which resulted in the final nested case-control study cohort of 302 case-control pairs.

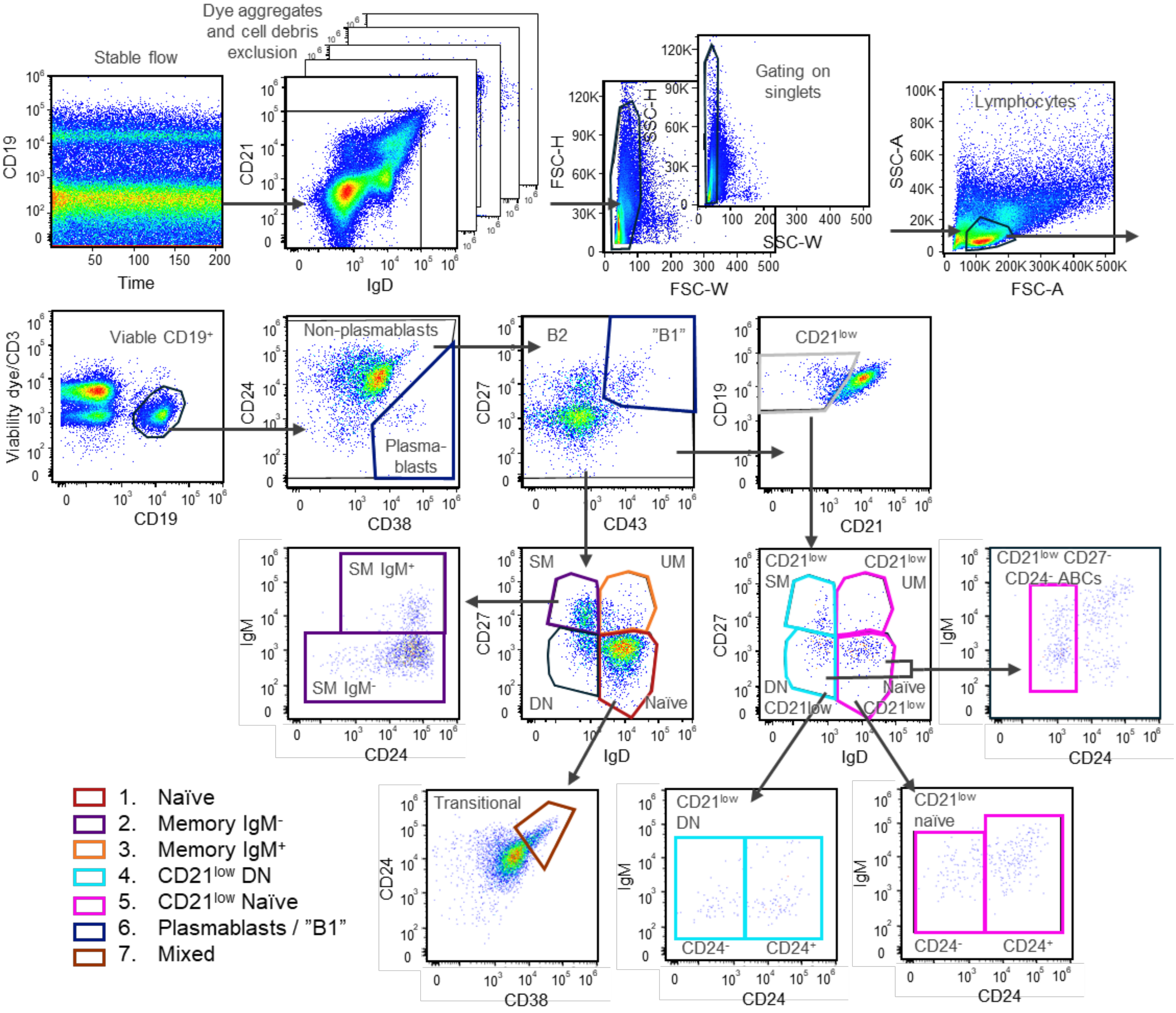

Supplemental Figure 2. Gating of B-cell populations in human PBMC samples (representative of n=1) from the Malmö Diet and Cancer study. The gating strategy was based on publications by Maecker et al., 2012 (DOI: 10.1038/nri3158) and Griffin et al., 2011 (DOI:10.1084/jem.20101499). First, cells were gated on time to remove artefacts due to unstable flow followed by removal of dye aggregates and cell duplets. Lymphocytes were identified on forward vs side scatter plots and viable total B cells as viable $CD19^+CD3^-$ cells. First, $CD38^+CD24^-$ plasmablasts were gated, followed by $CD43^+CD27^+$ "B1"-cells. From the remaining "B2"-cells IgD^+CD27^- naïve, IgD^+CD27^+ unswitched memory (UM), IgD^-CD27^+ switched memory (SM) and IgD^-CD27^- double negative (DN) cells were identified. From each of these 5 populations $CD21^{low}$ cells were identified on CD21 vs CD19 plots. The SM population was further gated on CD24 vs IgM to identify IgM^+ and IgM^- SM cells. The naïve population was gated on CD38 vs CD24 to identify $CD38^+CD24^+$ transitional B-cells. From the B2-cells total $CD21^{low}$ cells were then identified on CD21 vs CD19 plots, and subsequently $CD21^{low}$ naïve, UM, SM and DN cells on IgD vs CD27 plots. The $CD21^{low}$ DN and naïve cells were further gated to identify $CD24^+$ and $CD24^-$ cells.

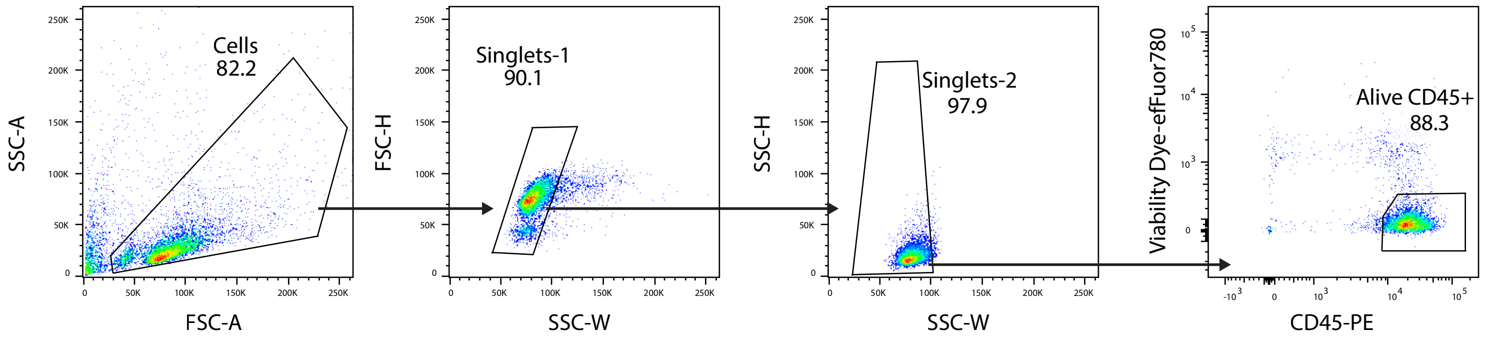

Supplemental Figure 3. Gating scheme of splenic CD45⁺-cells from Ldlr⁻/⁻ mice (representative of n=1) before single-cell RNA sequencing. Gating strategy of alive splenic CD45⁺-cells for sorting from young and aged chow diet-fed female Ldlr⁻/⁻ mice.

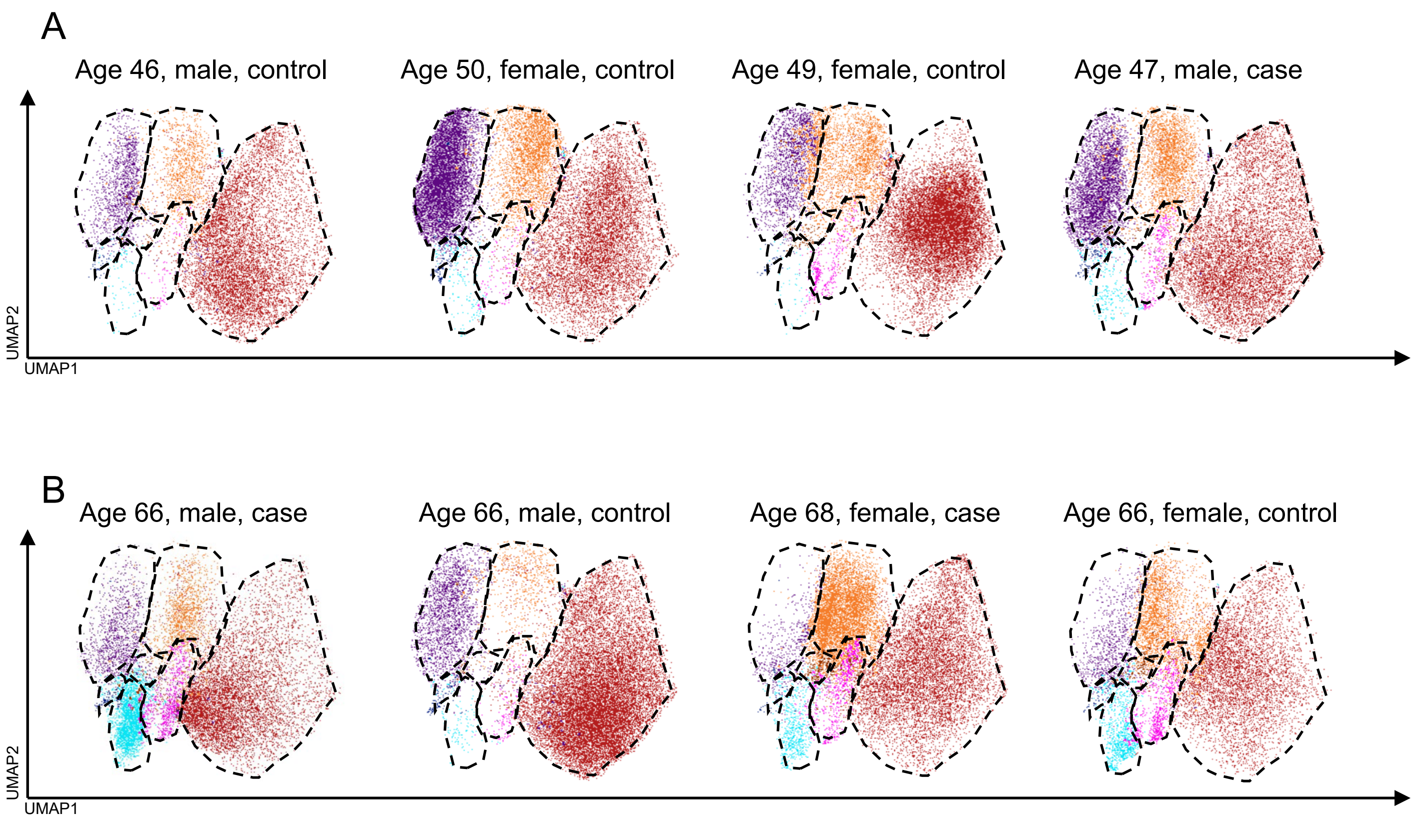

Supplemental Figure 4. Mapping of B-cell populations in middle-aged and old individuals. Unsupervised clustering combined with UMAP visualization of major B-cell populations in flow cytometry data from **A:** 4 middle-aged (≤50 years old) individuals and **B:** 4 old (≥66 years old) individuals to exemplify individual variations.

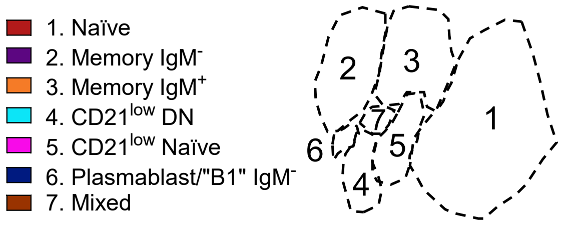

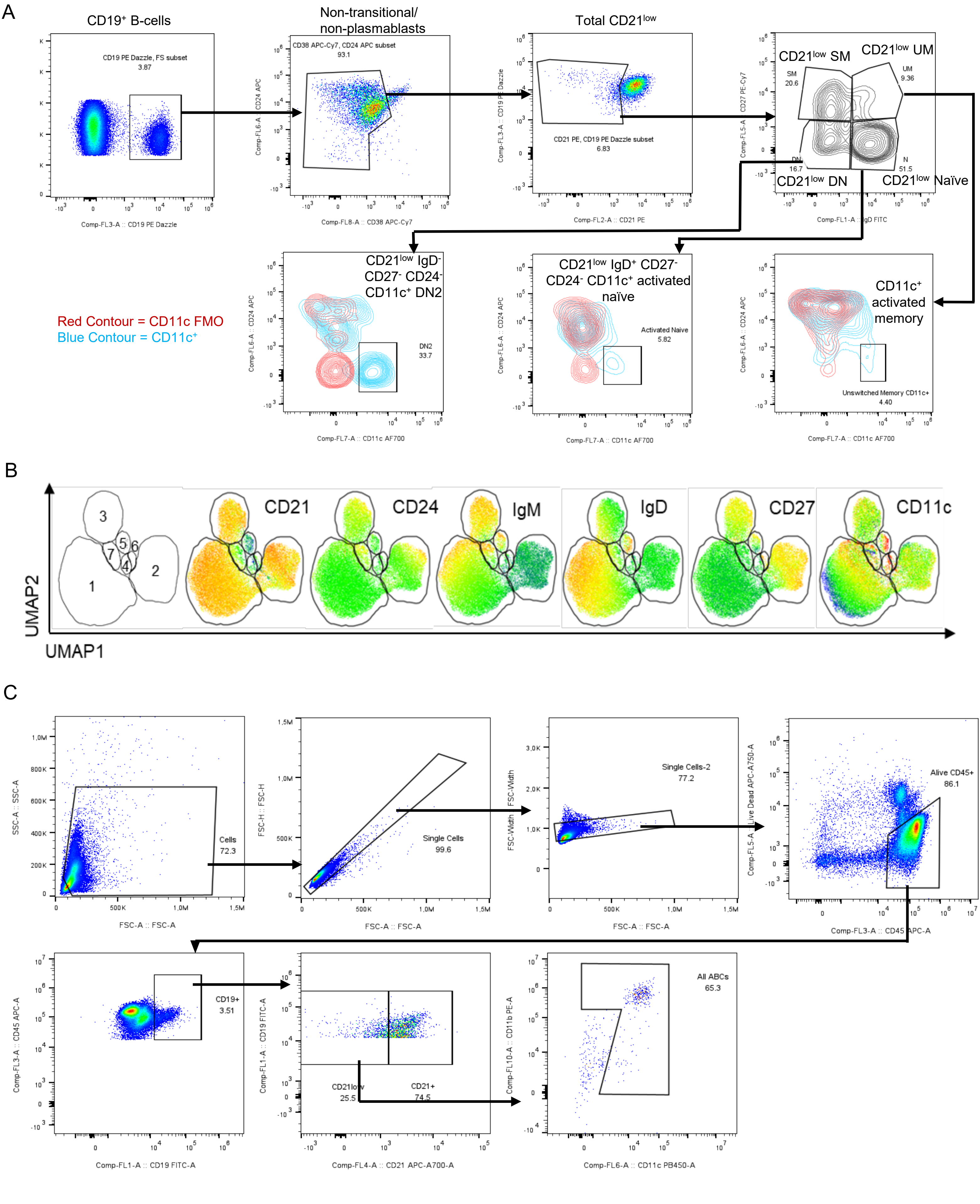

Supplemental Figure 5. CD21low B-cells correspond to CD11c⁺ B-cell populations. **A:** CD21low IgD⁻ CD27⁻ DN cells (DN2) that are negative for CD24 are also CD11c⁺ and CD21low IgD⁺ CD27⁻ naïve cells (activated naïve) that are negative for CD24 are also CD11c⁺. CD11c gate boundaries were set with fluorescence minus one (FMO) controls. **B:** UMAP of human B-cell markers, confirming that confirms that low expression of CD21, CD27 and CD24 identifies B-cells that also express CD11c. **C:** Gating strategy of CD21low CD11b⁺/CD11c⁺ age-associated B-cells in PBMCs of carotid endarterectomy patients (n=9).

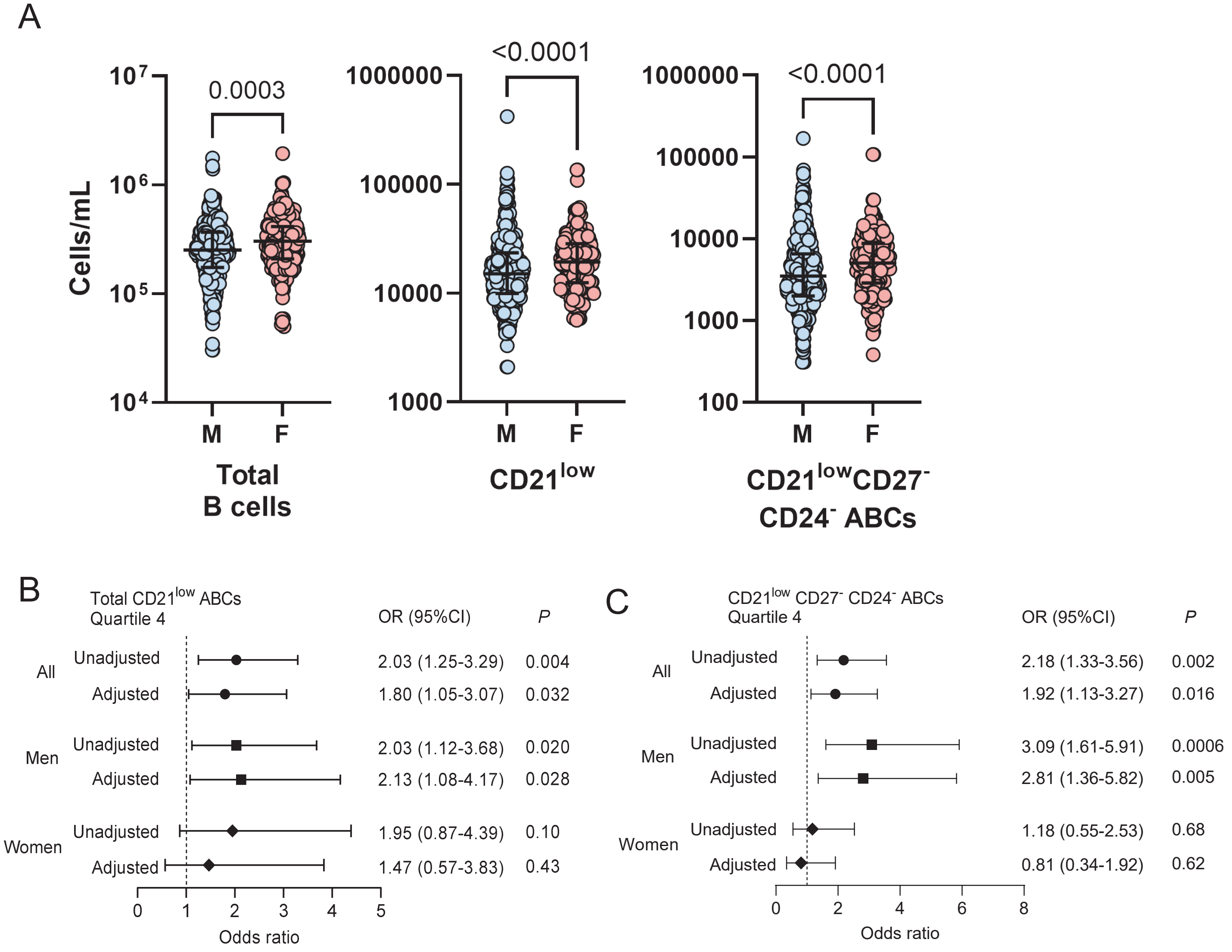

Supplemental Figure S6. A: Counts of total B-cells, CD21^{low} B-cells and CD21^{low}CD27$^-$CD24$^-$ ABCs between men (M) and women (F). Association between **B:** CD21^{low} B-cells and **C:** CD21^{low}CD27$^-$CD24$^-$ ABC counts and incident CE in men and women. Associations between quartiles of cell count and incident CE analyzed by conditional logistic regression in an unadjusted model (302 case-control pairs, 189 men and 113 women) and a model adjusted for CVD risk factors (total cholesterol, HDL, systolic blood pressure, diabetes, and current smoking; 279 case-control pairs, 147 men and 105 women). Odds ratios (OR) with 95% confidence intervals (CI) were compared to the lowest (reference) quartile.

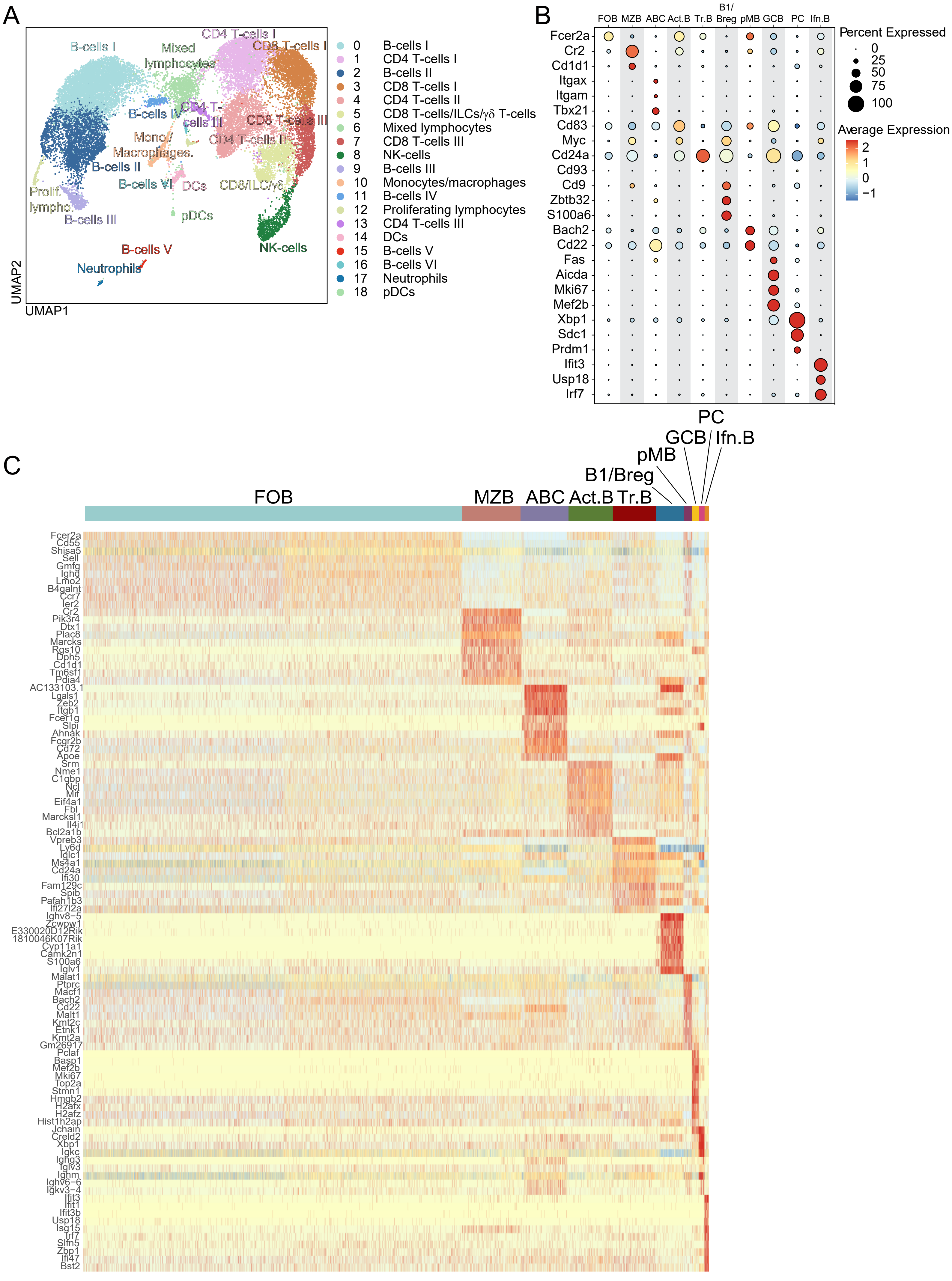

Supplemental Figure 7. Transcriptomic profiling of splenic CD45⁺-cells from young and old Ldlr$^{-/-}$ mice. **A:** UMAP visualization of clustered immune cells from spleens of young (pooled from n=7) and old (pooled from n=8) *Ldlr*$^{-/-}$ mice. **B:** Average expression of genes typical for each B-cell cluster. C: Heatmap of top 50 differentially expressed genes per B-cell cluster.

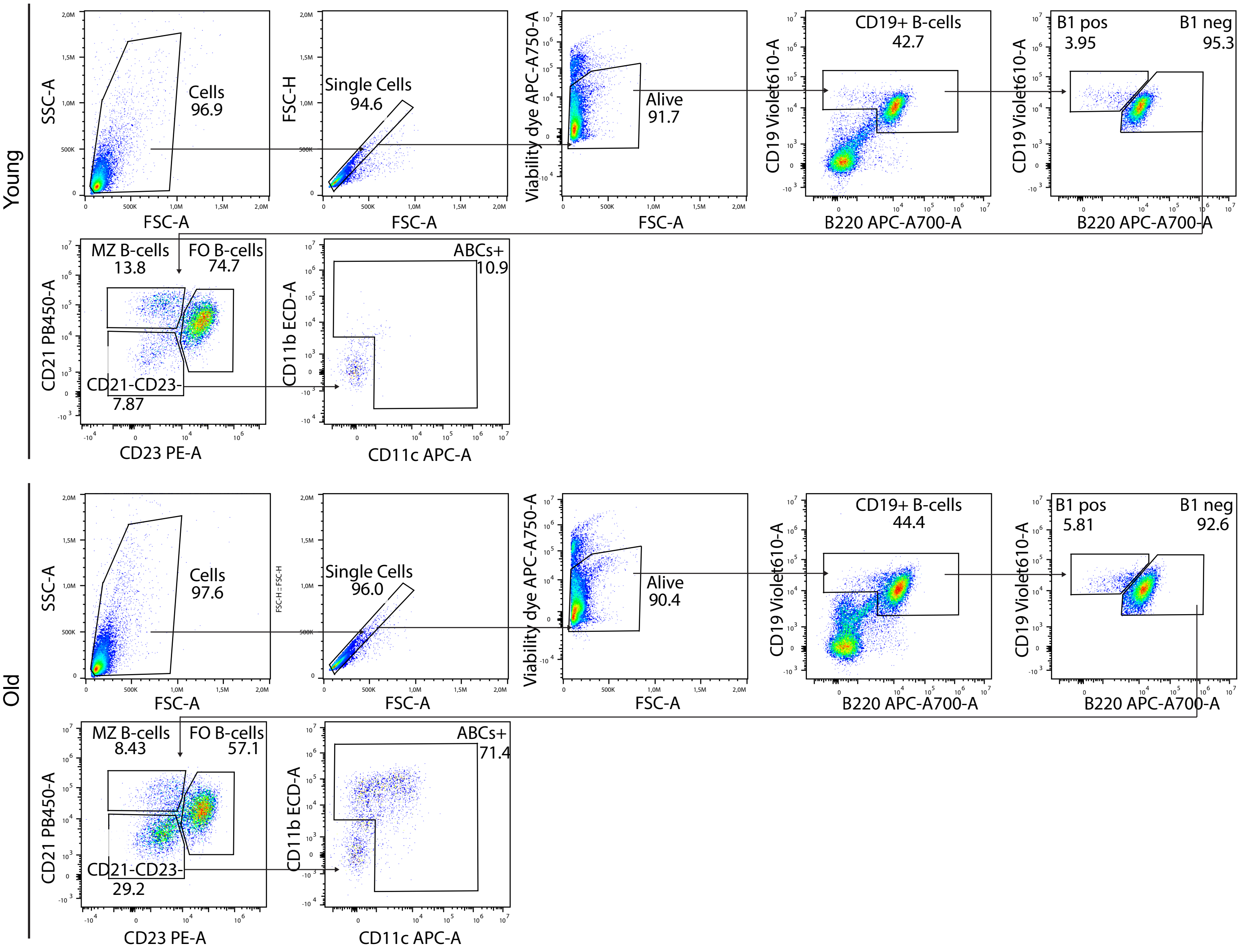

Supplemental Figure 8. Gating scheme of splenic B-cell subsets in *Ldlr*$^{-/-}$ mice. Gating strategy and representative plots of follicular B-cells, marginal zone B-cells, B1-cells and age-associated B-cells in spleens of young (representative of n=1) and aged (representative of n=1) chow diet-fed female *Ldlr*$^{-/-}$ mice.

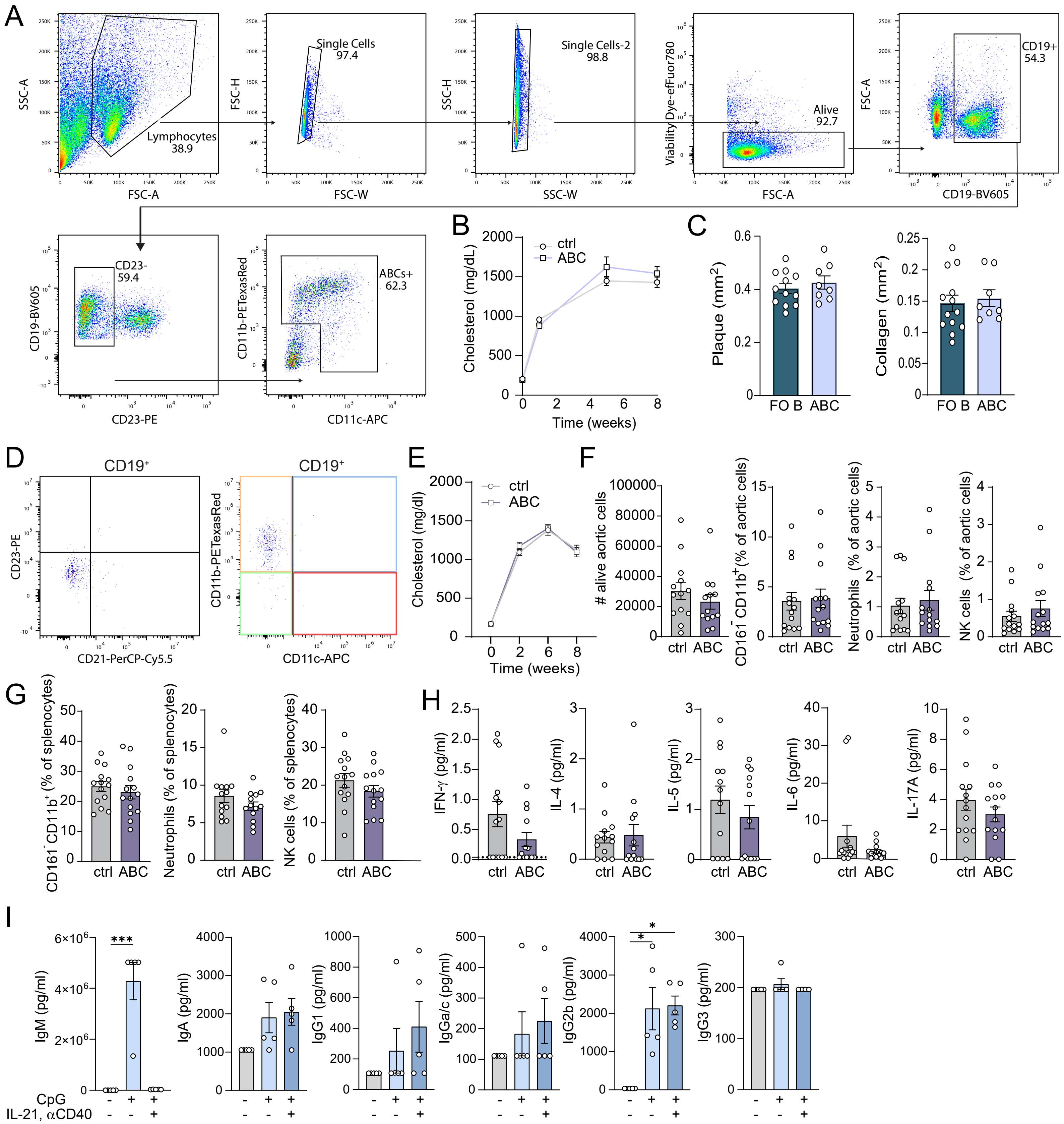

Supplemental Figure 9. Adoptive transfer of age-associated B-cells to *Ldlr*$^{-/-}$*Rag1*$^{-/-}$ mice. **A:** Gating strategy (representative of n=1) of CD19$^+$CD23$^-$CD11b$^+$ and/or CD11c$^+$ age-associated B-cells (ABCs) for sorting from spleens of aged (19-20 months) *Ldlr*$^{-/-}$ mice. **B:** Total cholesterol levels at sacrifice were measured in serum of control (n=8) and ABC-recipient (n=8) young female *Ldlr*$^{-/-}$ mice. **C:** Lesion size and collagen content of follicular B-cell (FOBs) (n=8)- and ABC-recipient (n=8) young female *Ldlr*$^{-/-}$ mice. Representative flow cytometry plot of **D:** CD21 and CD23 and CD11b and CD11c surface expression on peritoneal CD19$^+$ B cells in ABC recipient *Ldlr*$^{-/-}$*Rag1*$^{-/-}$ mice. **E:** Total cholesterol levels at sacrifice were measured in serum of control (n=13) and ABC-recipient (n=14) young female *Ldlr*$^{-/-}$*Rag1*$^{-/-}$ mice. **F:** Using flow cytometry, the number of alive cells, the percentage of CD161$^-$CD11b$^+$ myeloid cells, CD161$^-$CD11b$^+$Ly6C^{int}Ly6G$^+$neutrophils and CD161$^+$ NK-cells in the aortic root of control (n=13) and ABC-recipient (n=14) young female *Ldlr*$^{-/-}$*Rag1*$^{-/-}$ mice were measured. **G:** The percentage of CD161$^-$CD11b$^+$ myeloid cells, CD161$^-$CD11b$^+$Ly6C^{int}Ly6G$^+$neutrophils and CD161$^+$ NK cells in the spleen of control (n=13) and ABC-recipient (n=14) young female *Ldlr*$^{-/-}$*Rag1*$^{-/-}$ mice were measured with flow cytometry. **H:** Cytokine levels were quantified in serum of control (n=13) and ABC-recipient (n=14) young female *Ldlr*$^{-/-}$*Rag1*$^{-/-}$ mice using LEGENDplex. **I:** Measurement of immunoglobulins secreted by FOBs (n=5/condition) stimulated with TLR9-ligand CpG in a T cell-independent and T cell-dependent (IL-21, anti-CD40) setting. The dotted line indicates the lowest limit of detection. All replicates represent biological replictaes. Statistical significance was tested by two-tailed t-test or Mann-Whitney test. Mean ± S.E.M. plotted. *P < 0.05. ***P < 0.001.
