# Supplementary material for: Age-associated B cells predict coronary events in humans and aggravate murine atherosclerosis
Source: Cardiovasc Res. 2026 Jul 13;122(13):1789–802. doi: 10.1093/cvr/cvag154 (PMC13604844; doi:10.1093/cvr/cvag154)
Supplement: Supplementary file 1 [file cvag154_supplementary_data.zip › Suppmaterial.docx]

# **Supplemental material**

# Age-associated B-cells predict coronary events in humans and aggravate murine atherosclerosis

Jill de Mol, PhD.^1^*, Pernilla Katra, PhD^2^*, Virginia Smit, PhD^1^*, Anna Witteveen, MSc.^1^, Lukas Tomas, MD PhD^3,4^, Linda Andersson, MSc.^2^, Scott W.M. Engels, MSc.^1^, Samuel Andersson, MSc.^2^, Mireia N. A. Bernabé Kleijn, BSc.^1^, Alexandru Schiopu, MD PhD^5,6,7^, Eva Bengtsson, PhD^2,8,9^, Ilze Bot, Prof. PhD^1^, Daniel Engelbertsen, PhD^2^, Johan Kuiper, Prof. PhD^1^, Harry Björkbacka, PhD^2,#^, and Amanda C. Foks, PhD^1,#^

^1^Division of BioTherapeutics, Leiden Academic Centre for Drug Research, Leiden University, The Netherlands. ^2^Department of Clinical Sciences Malmö, Lund University, Sweden. ^3^Department of Medicine, LMU University Hospital, Munich, Germany. ^4^German Centre for Cardiovascular Research (DZHK), partner site Munich Heart Alliance, Munich, Germany. ^5^Department of Translational Medicine, Lund University, Malmö, Sweden. ^6^Department of Internal Medicine, Skåne University Hospital, Lund, Sweden. ^7^Nicolae Simionescu Institute of Cellular Biology and Pathology, Bucharest, Romania. ^8^Department of Biomedical Science, Malmö University, Malmö, Sweden. ^9^Biofilms – Research Center for Biointerfaces, Malmö University, Malmö, Sweden. *Authors contributed equally. #Authors share senior authorship

**Corresponding author**: Amanda C. Foks (a.c.foks@lacdr.leidenuniv.nl), Division of BioTherapeutics, Leiden University, LACDR, Einsteinweg 55, 2333CC Leiden, The Netherlands.

## Content

### 1. Supplemental Methods

### 2. Supplemental Tables

### 3. Supplemental Figures

## 1. Supplemental Methods

### Human cohort study design

The Malmö Diet and Cancer (MDC) study is a prospective population-based cohort, based on the population of Malmö between 45-73 years old (Figure S1). During the years 1991-1996, 28 449 participants were included in the study. Between October 1991 and February 1994 every other included participant was also invited to a sub-study focused on cardiovascular disease, creating a cohort of 6103 participants (MDC-CV). All study participants were followed from study inclusion until first hospitalisation for a CE, death, emigration, or until June 30, 2009. A CE was defined as fatal or non-fatal myocardial infarction or death from ischemic heart disease, according to International Classification of Diseases, Ninth Revision (ICD9) codes 410, 412, 414 and ICD10 codes I21, I22, I23, and I25.

During the follow-up period, 491 incident CE cases (8.0% of the MDC-CV cohort) were retrieved by data linkage to the Swedish Hospital Discharge Register and the Cause of Death Registry of Sweden. 36 participants with a prevalent CE before study inclusion were then excluded, as well as 87 participants who lacked a PBMC sample or could not be matched to a control. The remaining 368 participants with an incident CE were matched to a control using incidence density sampling based on age (± 1 year) and sex, resulting in a nested case-control cohort of 368 matched case-control pairs. After flow cytometric analysis of the cohort 66 case-control pairs had to be excluded due to poor sample quality after thawing, low cell counts or non-adherence to the standard operating procedures which resulted in the final cohort used for statistical analysis of 302 case-control pairs.

The study was approved by the regional ethics review board (LU 51-90 and LU 532-2006) and was conducted in accordance with the Declaration of Helsinki. All participants gave written informed consent.

### Laboratory analyses

At the study baseline, participants underwent a physical examination, and answered a questionnaire regarding lifestyle and medical history. Blood was drawn after an overnight fast for analysis of glucose, HbA1c, triglycerides, cholesterol, high-density lipoprotein (HDL), white blood cells (WBC) and lymphocytes using standard methods at the Department of Clinical Chemistry at Malmö University Hospital. Low-density lipoprotein (LDL) was calculated with Friedewald’s formula. Diabetes was defined as fasting whole blood glucose >6.1 mmol/L (corresponding to a threshold of 7.0 mmol/L in fasting plasma glucose), self‐reported physician diagnosis of diabetes mellitus, or use of anti-diabetic medication. The estimated glomerular filtration rate (eGFR) was calculated using the MRDR formula.

### Flow cytometry analysis of human PBMCs

Blood was collected in heparinised tubes and PBMCs were isolated using density gradient centrifugation (Lymphoprep, STEMCELL Technologies; Ficoll-Paque PLUS, GE Healthcare Life Sciences). PBMCs were frozen slowly in equal volumes of autologous serum and RPMI-1640 (Thermo Fisher Scientific) containing 20% DMSO (Sigma-Aldrich), by first being placed in a Styrofoam box at -80°C for at least one hour and then transferred to -140°C for long-term preservation.

PBMC processing and flow cytometry staining was performed as described previously, as well as the set-up, calibration and daily quality assurance of the 3 laser, 10-color Gallios flow cytometer (Beckman Coulter) that the samples were acquired on^1^. The antibodies used in the study are presented in Supplemental Table S1. Only one lot of each antibody was used throughout the study. Single-stained compensation beads (BD CompBeads anti-mouse Igκ, BD Biosciences) were used for calculating the compensation. The flow cytometry data was analysed in FlowJo v10.5, quality assurance was performed in Kaluza Analysis v1.3 (Beckman Coulter).

Unsupervised clustering and nonlinear dimensionality reduction via UMAP visualisation was performed in FlowJo 10.10.0 using plugins FlowSOM v3.0.18^2^, UMAP v3^3^. and Cluster Explorer as described by Ujas *et al.*^4^ The analysis was performed using concatenated live CD3^-^CD19^+^ B-cells from 8 individuals, 4 older, ≥66 years old, and 4 younger, ≤50 years old, (4 men and 4 women, 2 CE cases and 5 controls and 1 case and control, with CD19^+^ B-cell counts close to the median). There was a similar number of flow cytometry events included in the analysis for both groups (64285 and 66829).

### Cardiovascular disease patient population

Human atherosclerotic plaques (n=9) and paired blood samples were obtained from patients undergoing a carotid endarterectomy procedure at the Haaglanden Medical Center, location Westeinde (The Hague, The Netherlands). The study was approved by the Leiden – The Hague – Delft Medical Ethical Committee (METC-LDD) (NL71516.058.19). The study was performed in accordance with the Declaration of Helsinki and all patients gave written informed consent at the start of the study. Single-cell suspensions of human peripheral blood mononuclear cells (PBMCs) and carotid plaques were obtained as described previously^5^, and stained with Fix Viability Dye eFluor^TM^ 780 (1:2000, eBioscience), CD45-APC (2D1, 1:20, Biolegend), CD19-FITC (HIB19, 1:20, Biolegend), CD21-AF700 (Bu32, 1:20, Biolegend), CD11b-PE (Bear1, 1:20, Biolegend) and CD11c-BV421 (3.9, 1:20, Biolegend).

### Animals

All animal work was approved by local animal ethics committees and executed in line with governmental guidelines and the guidelines postulated in Directive 2010/63/EU of the European Parliament on the protection of animals used for scientific purposes. Female *Ldlr^-/-^* and *Ldlr^-/-^ Rag1^-/-^* mice (young: 2-5 months, aged: 18-22 months) on a C57BL/6J genetic background were bred and aged in-house. *Tcrα^-/-^* (B6.129S2-Tcratm1Mom/J) mice were purchased from Jackson Laboratories and crossed with *Ldlr^-/-^* mice in-house. Female *Tcrα^-/-^ Ldlr^-/-^* mice were aged for 44-51 weeks. Mice were fed a regular chow diet (CD) or Western-diet (WD) containing 0.25% cholesterol and 15% cocoa butter (Special Diet Services, Witham, Essex, UK) and kept under standard laboratory conditions. Diet and water were provided *ad libitum*. At the end of experiment, mice were anesthetized by a subcutaneous injection containing a cocktail of ketamine (100 mg/kg), atropine (0.5 mg/kg) and xylazine (10 mg/kg) and mice were sacrificed by retro-orbital bleeding. One mouse was excluded from the experiment due to hydronephrosis.

### Cell suspensions

Aortic arch, spleen, mediastinal lymph nodes (LN), blood, perigonadal white adipose tissue (WAT), peritoneal cells (Per), and bone marrow (BM) were harvested after *in situ* perfusion with PBS. BM cells were obtained by flushing the tibia and femora with PBS. Single-cell suspensions of spleen, BM and LN were obtained by mashing the organs through a 70 μm cell strainer. Erythrocytes were removed from splenocytes and blood by lysing samples for 2 minutes with ACK lysis buffer (NH_4_Cl 150 mM, KHCO_3_ 10 mM, Na_2_EDTA 0.1mM, pH 7.3). Aortic arches were cleaned from residual adipose tissue and enzymatically digested as described previously^5^. WAT was digested with collagenase I (450 U/mL) and collagenase XI (250 U/mL) for 45 min at 37 °C, and subsequently mashed through 70 μm cell strainers. Single-cell suspensions were analyzed by flow cytometry.

### Flow cytometry of murine B-cells

Single-cell suspensions were either extracellularly stained with anti-mouse fluorochrome-conjugated antibodies for 30 min at 4 °C, in which live/dead viability staining with Fixable Viability Dye eFluor™ 780 (1:2000, eBioscience) was used for exclusion of non-viable cells; or extracellularly stained with Fixable Live/dead Zombie Aqua for 10 min at 4°C, after which extracellular anti-mouse fluorochrome-conjugated antibodies were added for another 20 min. Fc block was used to block Fc receptors to prevent aspecific binding of antibodies. Prior to intracellular staining, cells were fixed and permeabilized according to manufacturer's protocol (Invitrogen, transcription factor: Transcription factor FoxP3 Buffer Set, BD Biosciences). After fixation and permeabilization, cells were intracellularly stained for 30 min at 4 °C. All antibodies used for flow cytometry are listed in Table S2. Flow cytometry measurements were performed on a CytoFLEX S (Beckman Coulter, USA). Data were analyzed using FlowJo v10.7 software (Treestar).

*Immunofluorescent immunological staining of murine spleen*

The spleen of an aged (20 months) female *Ldlr^-/-^* mouse was harvested, embedded in O.C.T. compound (Sakura) and snap-frozen. Cryosections of 10 µm were collected and blocked with 5% milk buffer for 1h and stained with CD19-FITC (MB19.1, Biolegend, 1:200), CD3e-APC (1452C11, Biolegend, 1:200) and CD11c-PE (N418, eBioscience, 1:200) overnight. Sections were mounted with DAPI fluorshield, scanned using a Pannoramic 250 (3DHISTECH) at magnification 20, and analyzed using QuPath.

### Splenic CD45^+^ enrichment for single-cell RNA sequencing

Single-cell splenocyte suspensions were obtained from CD-fed female *Ldlr^-/-^* mice (young: 5 months, aged: 21 months) as described above. Splenocytes were pooled per age group (young: n=7, aged: n=8), blocked with Mouse TruStain FcX™ and stained with Fixable Viability Dye-eFluor 780, CD45-PE, and TotalSeq-C antibodies listed in Tables S2 and S34. After doublet removal, alive CD45^+^ cells were sorted (Figure S3) in PBS supplemented with 0.04% BSA using the FACS Aria II SORP (BD Biosciences) and immediately processed for single-cell RNA sequencing (scRNA-seq).

### Single-cell RNA sequencing of CD45^+^ splenocytes

Splenic leukocyte suspensions were loaded into a 10x Chromium Single Cell Controller (10x Genomics) along with gel beads, partitioning oil, and reverse transcription reagents to generate single-cell gel bead-in-emulsions (GEMs). ScRNA-seq libraries were prepared using the Next GEM Single Cell 5’ Kit V2 (10x Genomics). Concomitantly, scBCR-seq libraries were prepared by using the Single Cell V(D)J Enrichment Kit, Mouse B-cell (10x Genomics). All samples were sequenced with an Illumina HiSeq2500 sequencer to achieve library construction. Digital expression matrixes were generated by de-multiplexing the barcodes of each cell and the unique molecular identifier (UMI) of all transcripts by using the Cell Ranger v6.0 pipeline (10x Genomics).

### Single-cell RNA-sequencing data processing and analysis

The generated expression matrixes were loaded into R and analysed with the R package Seurat^6^. Quality of cells was ensured by filtering cells based on the unique gene count per cell (young, >200 and < 3500: aged, >200 and <4000), percent of mitochondrial genes of all genes (<6%), and ribosomal gene expression (young, <25%: aged, <22%). Filtered cells were subsequently log-normalized and the gene space was scaled accordingly. Moreover, doublets were removed by using the DoubletFinder R package^7^, discarding 920 young cells and 644 aged cells and the remaining 13540 young cells and 10042 aged cells were used for subsequent analysis. After doublet removal, the V(D)J sequences of BCRs were loaded into R and coupled to their corresponding B-cell by using the R package scRepertoire^61^. Clonotypes were subdivided into four categories depending on their frequency in cells: 1) single (0< cells ≤1), 2) small (1< cells ≤5), 3) medium (5< cells ≤20), and 4) large (20< cells ≤100). Young and aged cells were subsequently merged, log-normalized and the top 2000 variable features within each age group were determined. Genes that encode for the V(D)J sequences of TCRs and BCRs were discarded as variable features. Hereafter, features that were repeatedly variable across both age groups were identified and used as immune anchor for the integration of young and aged cells. Principal component analysis (PCA) was performed on a total of 23582 cells. 12 PCA components were included for cluster detection at a resolution of 0.46. Clusters were projected via Uniform Manifold Approximation and Projection (UMAP) and annotated based on the canonical marker genes and differentially expressed genes per cluster (Table S6), which were identified via the Seurat function FindAllMarkers(). Expression of several genes was validated on protein level by the TotalSeq-C antibodies listed in Table S3. For the B-cell subclustering, *Cd79a*^+^ B-cells clusters were selected from the main clustering. To exclude non-B-cells, thresholds of genes were set: Thy1<0.3 & Cd3e<0.3 & Cd3d<0.3 & Cd3g<0.3 & Cd4<0.3 & Cd8a<0.3 & Cd8b1<0.3 & Cd79a>0.3 & Cd79b>0.3. The variable genes of selected B-cells were identified, log-normalized, rescaled, and subsequently used for dimensionality reduction on 8588 B-cells. 17 PCA components were used for cluster detection at a resolution of 0.2. Clusters were projected via UMAP and annotated based on the differentially expressed genes per cluster (Table S7) as previously described. The clonality of BCRs was analysed with the R package scRepertoire^8^. To evaluate clonal diversity between young and aged mice, the Shannon entropy and Inverse Simpson Index, both measures of clonal richness and population structure, were determined^9^. As these formulas measure the diversity in a population, low diversity will result in low Shannon and Inverse Simpson indices. The proportion of overlapping clones was measured by the Morisita-Horn index, which assigns 0 and 1 values to unrelated and identical clonotypes, respectively.

### Adoptive transfer of ABCs

Single-cell splenocyte suspensions were obtained from CD-fed female *Ldlr^-/-^* mice (18-21 months old) and stained with Fixable Viability Dye-eFluor780, CD19-BV605, CD11b-PEDazzle594, CD11c-APC and CD23-PE (Supplemental Table S2). Alive, singlet age-associated B-cells (CD19^+^ CD23^-^ CD11b^+^/CD11c^+^) were sorted using a FACS Aria II SORP (BD Biosciences) with an 85 µm nozzle in PBS for adoptive transfer into female *Ldlr^-/-^* (n=8/group) or *Ldlr^-/-^Rag1^-/-^* mice (n=13-14/group) (2-3 months old). Sorted ABCs were stained with CD21-BV421 to confirm low expression of CD21. Mice were randomized according to basal weight and total serum cholesterol levels. From the start of the experiment (T=0 weeks), mice were fed a WD for 8 weeks. Mice received intravenous injections of 5×10^5^ FACS-sorted ABCs or PBS (control) at T=0 and T=4 weeks. During the experiment, blood was collected by tail vein bleeding on T=0, T=2 and T=6 weeks, and by retro-orbital bleeding at sacrifice on T=8 weeks.

### Adoptive transfer of follicular (FO) B-cells

Single-cell splenocyte suspensions were obtained from CD-fed female *Ldlr^-/-^* mice (18-21 months old) and stained with Fixable Viability Dye-eFluor780, CD19-BV605, CD11b-PEDazzle594, CD11c-APC, CD23-PE and CD21-BV421 (Supplemental Table S3). Alive, singlet follicular B-cells (CD19^+^ CD23^+^ CD21^int^) were sorted using a FACS Aria II SORP (BD Biosciences) with an 85 µm nozzle in PBS for adoptive transfer into female *Ldlr^-/-^* mice (n=8/group) (2-3 months old). Mice were randomized according to basal weight and total serum cholesterol levels. From the start of the experiment (T=0 weeks), mice were fed a WD for 8 weeks. Mice received intravenous injections of 5×10^5^ FACS-sorted follicular B-cells at T=0 and T=4 weeks. During the experiment, blood was collected by tail vein bleeding on T=0, T=2 and T=6 weeks, and by retro-orbital bleeding at sacrifice on T=8 weeks.

### Histology

Hearts were embedded in O.C.T. compound (Sakura) and snap-frozen. To determine lesion size, cryosections (5 µm) of the aortic root were stained with Oil-Red-O and hematoxylin (Sigma-Aldrich). The average of five sequential sections of the three-valve area displaying the highest lesion content were used to measure the lesion size (mm^2^). Sections with the largest plaque in the three-valve area plus two flanking sections were analysed for plaque composition. Collagen content in the lesions was quantified using a Masson’s trichrome staining (Sigma-Aldrich). The necrotic core was defined as the acellular, debris-rich lesion area as percentage of total plaque area. Corresponding sections on separate slides were stained for monocyte/macrophage content with a rat IgG2b MOMA-2 antibody (1:1000, MCA519G, AbD Serotec), followed by a biotinylated rabbit ant-rat IgG antibody (1:200, BA-4001, Vector Laboratories). Secondary antibodies were detected using the Vectastain ABC kit (Vector) and visualized with ImmPACT NovaRED HRP substrate (Vector). The relative amount of macrophages, collagen, and necrotic core in the plaque was determined as the percentage of total plaque area. Analysis and scoring were performed blinded. All sections were scanned using a Pannoramic 250 (3DHISTECH), captured using CaseViewer software (3DHISTECH), and manually analysed using ImageJ Fiji software (National Institutes of Health).

### Cholesterol assay

Serum was acquired via centrifugation and stored at -80°C for further analysis. Total cholesterol levels in serum were determined using enzymatic colorimetric procedures (Roche/Hitachi). Precipath (Roche/Hitachi) was used as an internal standard.

### LEGENDplex assay

Serum was acquired via centrifugation and stored at -80°C for further analysis. Immunoglobulin concentrations were measured in serum and supernatant from the stimulation assay using LEGENDplex™ Immunoglobulin Isotyping Panel assay according to manufacturer's instructions. Cytokine levels in serum were measured with LEGENDplex™ MU Th Cytokine Panel (Biolegend) according to manufacturer's instructions. LEGENDplex™ measurements were performed on a CytoFLEX S (Beckman Coulter, USA).

### Stimulation assay ABCs

FACS-sorted ABCs or follicular (FO) B-cells were cultured at 37°C, 5% CO_2_ in complete medium (RPMI 1640 containing 5% heat-inactivated FBS, L-glutamine, 100 IU/ml penicillin, 100 µg/ml streptomycin and 0.05 mM β-mercaptoethanol) in the absence or presence of 0.5 µM CpG (ODN-1826, InvivoGen). To simulate T-cell stimulation, B-cells were exposed to 25 ng/ml IL-21, 10 µg/ml anti-CD40 and 0.5 µM CpG. FO B-cells and ABCs were cultured in quintuplicate for 72h, after which the supernatant was stored at -80°C for further analysis. Immunoglobulin concentrations were measured using LEGENDplex™ as described in the Supplemental material.

*Plasma cell proliferation assay*

CD19^+^ B-cells were isolated from the spleens of aged (20 months, n=5) female *Ldlr^-/-^* mice using Mojosort Magnetic cell separation CD19 Nanobeads (Biolegend). 50.000 CD19^+^ B-cells were cultured in triplo at 37°C, 5% CO_2_ in complete medium (RPMI 1640 containing 10% heat-inactivated FBS, 1% L-glutamine, 100 IU/ml penicillin, 100 µg/ml streptomycin and 0.05 mM β-mercaptoethanol) in the absence or presence of 25 ng/ml IL-21, 100 ng/ml CD40L, and/or 1 µg/mL CpG (ODN-1826, InvivoGen) and 1 µg/mL anti-IgM. After 6 days, cells were harvested and the expression of ABC and plasma cell markers (Supplemental Table S5) were measured using a Northern Lights Spectral Flow Cytometer (Cytek Biosciences).

### Statistics

Statistical analyses on human data were performed in IBM SPSS Statistics 27. Figures were made in GraphPad Prism 9.1 and Cluster Explorer (FlowJo 10.10.0). Baseline data is presented as median with interquartile range (IQR) if continuous or count (percent of the total) if categorical. Differences in baseline measurements between cases and controls were analyzed with the Mann-Whitney or χ^2^ test. Associations between B-cell populations (cell counts divided into quartiles) and incident coronary events were explored using conditional logistic regression conditioned for the matched case-control pairs. Quartile 1 with the lowest values of B-cells was used as the reference. The regression models (matched for age and sex) were unadjusted or adjusted for CVD risk factors (total cholesterol, HDL, diabetes, systolic BP and current smoking). HDL values were natural logarithm transformed due to their non-normal distribution.

## Data of the murine experiments are reported as mean ± standard error of the mean (SEM). Outliers were identified and removed using Grubbs outlier test (a=0.05). Normally distributed data of two groups were tested for significance using an unpaired t-test. Data from more than two groups were analyzed by a one-way ANOVA. If data were not normally distributed, two groups were compared using a Mann-Whitney test, and three or more groups were compared via a Kruskal-Wallis test. P-values of <0.05 were considered significant. Statistical analyses were performed using GraphPad Prism 9.0 (GraphPad Software).

*References*

1. Tomas L, Katra P, Badn W, Andersson L, Nilsson J, Schiopu A, Engelbertsen D, Gonçalves I, Bengtsson E, Björkbacka H (2023) Invariant natural killer T cells and incidence of first-time coronary events: a nested case-control study. European Heart Journal Open. https://doi.org/10.1093/EHJOPEN/OEAD094
2. Van Gassen S, Callebaut B, Van Helden MJ, Lambrecht BN, Demeester P, Dhaene T, Saeys Y (2015) FlowSOM: Using self-organizing maps for visualization and interpretation of cytometry data. Cytometry A 87:636–645
3. McInnes L, Healy J, Melville J (2018) UMAP: Uniform Manifold Approximation and Projection for Dimension Reduction.
4. Ujas TA, Obregon-Perko V, Stowe AM (2023) A Guide on Analyzing Flow Cytometry Data Using Clustering Methods and Nonlinear Dimensionality Reduction (tSNE or UMAP). Methods Mol Biol 2616:231–249
5. Smit V, De Mol J, Schaftenaar FH, et al (2023) Single-cell profiling reveals age-associated immunity in atherosclerosis. Cardiovasc Res 119:2508–2521
6. McGinnis CS, Murrow LM, Gartner ZJ (2019) DoubletFinder: Doublet Detection in Single-Cell RNA Sequencing Data Using Artificial Nearest Neighbors. Cell Syst 8:329-337.e4
7. Borcherding N, Bormann NL, Kraus G (2020) scRepertoire: An R-based toolkit for single-cell immune receptor analysis. F1000Res. https://doi.org/10.12688/F1000RESEARCH.22139.2/DOI
8. Maecker HT, McCoy JP, Nussenblatt R (2012) Standardizing immunophenotyping for the Human Immunology Project. Nat Rev Immunol 12:191–200
9. Rempala GA, Seweryn M (2013) Methods for diversity and overlap analysis in T-cell receptor populations. J Math Biol 67:1339–1368

## 2. Supplemental Tables

**Supplemental Table S1: Malmö Diet and Cancer study B-cell antibody panel**

| **Marker** | **Fluorochrome** | **Clone** | **Cat. No.** | **Company** |
| --- | --- | --- | --- | --- |
| IgD | FITC | IADB6 | - | Beckman Coulter |
| CD21 | PE | BL13 | - | Beckman Coulter |
| CD19 | ECD | J3-119 | - | Beckman Coulter |
| CD27 | PE/Cy7 | 1A4CD27 | - | Beckman Coulter |
| CD24 | APC | ALB9 | - | Beckman Coulter |
| CD38 | APC-AF700 | LS198-4-3 | - | Beckman Coulter |
| CD43 | APC-AF750 | DFT1 | - | Beckman Coulter |
| IgM | PB | SA-DA-4 | - | Beckman Coulter |
| Zombie Aqua™ Fixable Viability Dye | - | - | 423102 | BioLegend |
| CD3 | KrOrange | UCHT1 | B00068 | Beckman Coulter |

**Supplemental Table S2. Antibodies for flow cytometry of murine B-cells**

| **Marker** | **Fluorochrome** | **Clone** | **Cat. No.** | **Working concentration** | **Source** |
| --- | --- | --- | --- | --- | --- |
| CD45 | AF700 | 30-F11 | 103128 | 1:1000 | Biolegend |
| CD11b | BV605 | M1/70 | 101257 | 1:500 | Biolegend |
| Ly6C | APC | HK1.4 | 17-5932-82 | 1:500 | eBioscience |
| Ly6G | PerCP | 1A8 | 127654 | 1:500 | Biolegend |
| CD45 | PE | 30-F11 | 103106 | 1:500 | Biolegend |
| CD19 | BV605 | 6D5 | 115540 | 1:500 | Biolegend |
| B220 | AF700 | RA3-6B2 | 103232 | 1:800 | Biolegend |
| CD11b | PEDazzle-594 | M1/70 | 101256 | 1:500 | Biolegend |
| CD11c | APC | N418 | 17-0114-82 | 1:1000 | eBioscience |
| CD21 | BV421 | 7E9 | 123422 | 1:400 | Biolegend |
| CD23 | PE | B3B4 | 101607 | 1:500 | Biolegend |
| CD138 | BV421 | 281-2 | 142508 | 1:500 | Biolegend |
| CD21 | PerCP-Cy5.5 | 7E9 | 123415 | 1:500 | Biolegend |
| CD11b | PE | M1/70 | 101208 | 1:1000 | Biolegend |
| CD161 | APC | PK136 | 17-5941-82 | 1:800 | eBioscience |
| Ly6C | PE-CF594 | HK1.4 | 562728 | 1:800 | BD Biosciences |
| CD16/32 (Fc Block) | - | 93 | 553142 | 1:250 | BD Biosciences |
| Fixable viability dye | eFluor-780 | - | 65-0865-18 | 1:2000 | eBioscience |

**Supplemental Table S3. TotalSeq-C antibodies**

| **TotalSeq-C antibody** | **Barcode Sequence** | **Cat. No.** | **Working concentration** | **Source** |
| --- | --- | --- | --- | --- |
| TotalSeq™-C0093 anti-mouse CD19 | ATCAGCCATGTCAGT | 115571 | 1:250 | Biolegend |
| TotalSeq™-C0107 anti-mouse CD21/CD35 (CR2/CR1) | GGATAATTTCGATCC | 123441 | 1:250 | Biolegend |
| TotalSeq™-C0108 anti-mouse CD23 | TCTCTTGGAAGATGA | 101639 | 1:250 | Biolegend |
| TotalSeq™-C0106 anti-mouse CD11c | GTTATGGACGCTTGC | 117361 | 1:250 | Biolegend |
| TotalSeq™-C0014 anti-mouse/human CD11b | TGAAGGCTCATTTGT | 101275 | 1:250 | Biolegend |
| TotalSeq™-C0110 anti-mouse CD43 | TTGGAGGGTTGTGCT | 143221 | 1:250 | Biolegend |
| TotalSeq™-C0117 anti-mouse I-A/I-E | GGTCACCAGTATGAT | 107659 | 1:250 | Biolegend |
| TotalSeq™-C0001 anti-mouse CD4 | AACAAGACCCTTGAG | 100571 | 1:250 | Biolegend |
| TotalSeq™-C0002 anti-mouse CD8a | TACCCGTAATAGCGT | 100785 | 1:250 | Biolegend |
| TotalSeq™-C0073 anti-mouse/human CD44 | TGGCTTCAGGTCCTA | 103063 | 1:250 | Biolegend |
| TotalSeq™-C0112 anti-mouse CD62L | TGGGCCTAAGTCATC | 104455 | 1:250 | Biolegend |
| TotalSeq™-C0078 anti-mouse CD49d | CGCTTGGACGCTTAA | 103633 | 1:250 | Biolegend |
| TotalSeq™-C0191 anti-mouse/rat/human CD27 | CAAGGTATGTCACTG | 124245 | 1:250 | Biolegend |
| TotalSeq™-C0204 anti-mouse CD28 | ATTAAGAGCGTGTTG | 102133 | 1:250 | Biolegend |
| TotalSeq™-C0004 anti-mouse CD279 (PD-1) | GAAAGTCAAAGCACT | 109127 | 1:250 | Biolegend |
| TotalSeq™-C0846 anti-mouse CD185 (CXCR5) | ACGTAGTCACCTAGT | 145537 | 1:250 | Biolegend |

**Supplemental Table S4. Antibodies for spectral flow cytometry of cultured murine B-cells.**

| **Marker** | **Fluorochrome** | **Clone** | **Cat. No.** | **Working concentration** | **Source** |
| --- | --- | --- | --- | --- | --- |
| CD21 | BV421 | 7E9 | 123422 | 1:2000 | Biolegend |
| CD138 | BV605 | 281-2 | 142515 | 1:2000 | Biolegend |
| CD23 | FITC | B3B4 | 405721 | 1:1000 | Biolegend |
| CD19 | PerCP-Cy5.5 | 1D3 | 152406 | 1:1000 | Biolegend |
| CD11c | PE | N418 | 117308 | 1:500 | Biolegend |
| B220 | PE-Cy7 | RA3-6B2 | 103222 | 1:500 | Biolegend |
| CD11b | APC | M1/70 | 17-0112-83 | 1:2000 | eBiocience |
| CD16/32 (Fc Block) | - | 93 | 553142 | 1:250 | BD Biosciences |
| Fixable viability dye | eFluor-780 | - | 65-0865-18 | 1:4000 | eBioscience |

**Supplemental Table S5: Spearman correlations of human B-cell populations and age.**

|  | **Spearman's Rho** | ***P*** | **N** |
| --- | --- | --- | --- |
| CD21^low^ B-cells | 0.13 | 0.001 | 604 |
| “B1” | -0.11 | 0.008 | 604 |
| UM | -0.17 | 0.000 | 604 |
| SM IgM^-^ | -0.20 | <0.001 | 604 |
| Total B-cells | -0.06 | 0.16 | 604 |

SM: IgD^-^CD27^+^ switched memory, UM: IgD^+^CD27^-^ unswitched memory. Spearman’s rank correlations were used to assess associations, and statistical significance was determined using corresponding p-values.

**Supplemental Table S6: Conditional logistic regression between CD21^low^ CD27^-^ CD24^-^ ABCs and inflammatory markers.**

|  |  | **Quartiles of CD21^low^ CD27^-^ CD24^-^ ABCs** | | | |
| --- | --- | --- | --- | --- | --- |
| **Model** | **Adjustments** | **1** | **2** | **3** | **4** |
| 1 | Unadjusted | Reference | 1.32 (0.82-2.14)*, *P*=0.25 | 1.47 (0.93-2.33), *P*=0.10 | 2.18 (1.33-3.56), *P*=0.002 |
| 2 | + Risk factors^#^ | Reference | 1.28 (0.76-2.15), *P*=0.36 | 1.29 (0.78-2.15), *P*=0.33 | 1.92 (1.13-3.27), *P*=0.016 |
| 3 | + Risk factors  + hsCRP | Reference | 1.37 (0.78-2.43), *P*=0.27 | 1.21 (0.70-2.09), *P*=0.51 | 1.80 (1.01-3.24), *P*=0.049 |
| 4 | + Risk factors  + WBC | Reference | 1.28 (0.76-2.15), *P*=0.36 | 1.29 (0.78-2.15), *P*=0.33 | 1.94 (1.13-3.33), *P*=0.017 |
| 5 | + Risk factors  + IL-6 | Reference | 1.61 (0.80-3.23), *P*=0.18 | 1.18 (0.62-2.24), *P*=0.62 | 3.03 (1.44-6.37), *P*=0.003 |

* Odds ratios (inter-quartile range).

^#^ Risk factors included total cholesterol, HDL, systolic blood pressure, diabetes, and current smoking. Statistical significances were analysed by conditional logistic regression conditioned for the matched case-control pairs.

**Supplemental Table S7: Conditional logistic regressions for human CD21^low^  B-cell subpopulations.**

|  | **B-cell quartile** | **Unadjusted*** | | **Adjusted^#^** | |
| --- | --- | --- | --- | --- | --- |
|  |  | **OR (95% CI)** | **p-Value** | **OR (95% CI)** | ***P*** |
| CD21^low^ IgD^-^ CD27^-^ | 1 | Reference | - | Reference | - |
|  | 2 | 1.23 (0.77-1.95) | 0.38 | 1.18 (0.71-1.96) | 0.53 |
|  | 3 | 1.10 (0.69-1.74) | 0.69 | 0.94 (0.57-1.56) | 0.81 |
|  | 4 | 1.97 (1.22-3.21) | **0.006** | 1.61 (0.94-2.74) | 0.08 |
| CD21^low^ IgD^+^ CD27^-^ | 1 | Reference | - | Reference | - |
|  | 2 | 0.76 (0.47-1.22) | 0.25 | 0.76 (0.44-1.31) | 0.33 |
|  | 3 | 1.46 (0.92-2.32) | 0.11 | 1.65 (0.98-2.77) | 0.06 |
|  | 4 | 1.50 (0.94-2.40) | 0.09 | 1.50 (0.88-2.55) | 0.13 |
| CD21^low^ IgD^-^ CD27^+^ | 1 | Reference | - | Reference | - |
|  | 2 | 1.05 (0.67-1.65) | 0.82 | 0.99 (0.60-1.64) | 0.98 |
|  | 3 | 1.17 (0.74-1.85) | 0.51 | 0.99 (0.60-1.66) | 0.98 |
|  | 4 | 1.51 (0.95-2.39) | 0.08 | 1.07 (0.63-1.83) | 0.80 |
| CD21^low^ IgD^+^ CD27^+^ | 1 | Reference | - | Reference | - |
|  | 2 | 1.16 (0.73-1.83) | 0.53 | 1.37 (0.82-2.29) | 0.22 |
|  | 3 | 1.27 (0.81-1.99) | 0.29 | 1.44 (0.87-2.39) | 0.16 |
|  | 4 | 1.09 (0.69-1.73) | 0.71 | 1.14 (0.68-1.90) | 0.63 |
| CD21^low^ IgD^-^ CD27^-^ CD24^+^ | 1 | Reference | - | Reference | - |
|  | 2 | 1.41 (0.89-2.23) | 0.145 | 1.24 (0.75-2.03) | 0.40 |
|  | 3 | 1.49 (0.95-2.34) | 0.080 | 1.36 (0.83-2.23) | 0.23 |
|  | 4 | 1.75 (1.08-2.84) | **0.024** | 1.43 (0.84-2.42) | 0.19 |
| CD21^low^ IgD^-^ CD27^-^ CD24^--^ | 1 | Reference | - | Reference | - |
|  | 2 | 1.2 (0.75-1.91) | 0.44 | 1.12 (0.67-1.88) | 0.67 |
|  | 3 | 1.53 (0.97-2.43) | 0.069 | 1.27 (0.76-2.1) | 0.36 |
|  | 4 | 1.72 (1.06-2.8) | **0.029** | 1.29 (0.75-2.22) | 0.35 |
| CD21^low^ IgD^+^ CD27^-^ CD24^+^ | 1 | Reference | - | Reference | - |
|  | 2 | 1.4 (0.89-2.19) | 0.15 | 0.63 (0.37-1.06) | 0.08 |
|  | 3 | 1.41 (0.87-2.28) | 0.16 | 0.9 (0.55-1.5) | 0.70 |
|  | 4 | 1.65 (1.03-2.64) | **0.036** | 0.92 (0.56-1.53) | 0.76 |
| CD21^low^ IgD^+^ CD27^-^ CD24^-^ | 1 | Reference | - | Reference | - |
|  | 2 | 1.29 (0.81-2.06) | 0.28 | 1.24 (0.74-2.07) | 0.41 |
|  | 3 | 1.97 (1.21-3.18) | **0.006** | 2.22 (1.30-3.80) | **0.004** |
|  | 4 | 1.87 (1.17-3.01) | **0.009** | 1.73 (1.03-2.91) | **0.038** |

*Pairs in unadjusted model: 302. # Pairs in adjusted model: 279. Statistical significances were analysed by conditional logistic regression conditioned for the matched case-control pairs.

**Supplemental Table S8: Counts of B populations in men and women**

| **B-cell population**  **(cells/mL blood)** | **Men**  **(N=378)** | **Women**  **(N=226)** | ***P*** |
| --- | --- | --- | --- |
| Total B-cells | 252200 (174100-367600) | 303200 (208300-414700) | **<0.001** |
| B2 cells | 242700 (166400-353500) | 282700 (198300-400700) | **<0.001** |
| “B1” cells (CD27^+^ CD43^+^) | 3700 (2200-5800) | 3700 (2300-5800) | 0.90 |
| Plasmablasts (CD38^+^ CD24^-^) | 1600 (900-2500) | 1500 (1000-2300) | 0.64 |
| Transitional B-cells | 11300 (7100-18500) | 14300 (8500-24100) | **<0.001** |
| Naïve B-cells (IgD^+^ CD27^-^) | 152800 (94000-241400) | 169600 (119100-277600) | **0.003** |
| Switched Memory (IgD^-^ CD27^+^) | 33600 (21100-57200) | 40900 (25600-72600) | **0.009** |
| - IgM^-^ Switched Memory | 27300 (16200-48900) | 29900 (15300-61700) | 0.08 |
| - IgM^+^ Switched Memory | 5000 (3000-8000) | 6200 (3700-12100) | **0.001** |
| Unswitched Memory (IgD^+^ CD27^+^) | 17500 (11200-26200) | 19400 (12600-30300) | **0.03** |
| Double Negative (IgD^-^ CD27^-^) | 19600 (12600-30700) | 24200 (16100-33300) | **<0.001** |
| CD21^low^ B-cells | 15000 (9900-23400) | 19300 (12600-28500) | **<0.001** |
| - CD21^low^ IgD^+^ CD27^-^ | 4900 (3000-9000) | 5800 (3700-9600) | **0.01** |
| - CD21^low^ IgD^+^ CD27^-^ CD24^-^ | 1900 (1000-3500) | 2200 (1100-4500) | **0.03** |
| - CD21^low^ IgD^+^ CD27^-^ CD24^+^ | 2800 (1700-4800) | 3500 (2300-5800) | **0.001** |
| - CD21^low^ IgD^-^ CD27^-^ | 3400 (2000-5500) | 4700 (3100-7600) | **<0.001** |
| - CD21^low^ IgD^-^ CD27^-^ CD24^-^ | 1400 (800-2700) | 2400 (1200-4100) | **<0.001** |
| - CD21^low^ IgD^-^ CD27^-^ CD24^+^ | 1600 (1000-2700) | 2100 (1400-3400) | **<0.001** |
| - CD21^low^ IgD^-^ CD27^+^ | 3500 (2000-5400) | 3900 (2700-7900) | **<0.001** |
| - CD21^low^ IgD^+^ CD27^+^ | 1800 (1000-3200) | 2100 (1100-3600) | 0.18 |
| ABCs (CD21^low^ CD27^-^ CD24^-^) | 3500 (2000-6500) | 5000 (2900-8800) | **<0.001** |

Statistical significances analysed by Mann-Whitney tests.

**Supplemental Table S9. Main clustering top 25 differentially expressed genes**

| **0. B-cells I** | **1. CD4 T-cells I** | **2. B-cells II** | **3. CD8 T-cells I** | **4. CD4 T-cells I** | **5. CD8 T /ILCs/gd T-cells** | **6. Mixed lymphocytes** |
| --- | --- | --- | --- | --- | --- | --- |
| Cd79a | Igfbp4 | Ly6d | Cd8b1 | Izumo1r | Ccl5 | Gm42418 |
| Cd74 | Ly6c1 | Ms4a1 | Cd8a | Tnfrsf4 | Gzmk | Malat1 |
| H2-Aa | Lef1 | Cd72 | Dapl1 | Ikzf2 | Nkg7 | AY036118 |
| H2-Eb1 | Tcf7 | Plac8 | Klk8 | Ctla4 | Ccl4 | mt-Cytb |
| Iglc2 | Actn1 | Cd79b | Saraf | Tigit | Cxcr6 | Lars2 |
| Ly6d | Rflnb | H2-Ab1 | Lef1 | S100a6 | Id2 | Sh3kbp1 |
| Cd79b | Lat | Cd79a | Dnajc15 | Lag3 | Ier3 | Mapre1 |
| Fcer2a | Npc2 | Unc93b1 | Fam241a | S100a4 | Ctla2a | Hnrnpm |
| H2-Ab1 | Nsg2 | Ifi30 | Rgcc | Cd5 | AW112010 | Dock2 |
| Iglc3 | Cd3d | Iglc2 | Npc2 | Cd3e | Rgs1 | Pik3cd |
| Fcmr | Cd4 | Mzb1 | Thy1 | Cd82 | Cxcr3 | Myh9 |
| H2-DMb2 | Cd3g | Cd74 | Cd3d | Cd4 | Hcst | Taf15 |
| Cd55 | Fxyd5 | Cd24a | Arl4c | Maf | Ms4a4b | Clint1 |
| H2-Ob | Cd247 | Napsa | Rplp0 | Tnfrsf18 | Ctsw | Actr2 |
| Ebf1 | Dgka | Ly86 | Igfbp4 | Cd3g | Il2rb | Pdcd4 |
| Ighd | Ccr7 | H2-Eb1 | Rplp1 | Pdcd1 | Cd3e | Ifi209 |
| Serpinb1a | Atp1b3 | Iglc3 | Eef1b2 | Tnfsf8 | Thy1 | Clk1 |
| Mef2c | Skap1 | Cd19 | Rpl12 | Hif1a | Cd3g | Hnrnpul2 |
| Tnfrsf13c | Cd5 | H2-DMb2 | Rpl5 | Icos | Eomes | Gm2a |
| Bank1 | Ms4a6b | Cr2 | Tcf7 | Itm2c | Lat | sep-09 |
| H2-Oa | Satb1 | H2-Aa | Actn1 | Ifi27l2a | Ctsd | Malt1 |
| Hvcn1 | Rgs10 | Pld4 | Fam189b | S100a11 | Xcl1 | Ppp1r15a |
| Ms4a1 | Ass1 | Blnk | Emb | Cd3d | Cd7 | Eif4a1 |
| Vpreb3 | Selenop | Fcmr | Il7r | Batf | S100a6 | Flna |
| Lmo2 | Slfn1 | Fcgr2b | Epsti1 | Itgb1 | Trbv13-2 | Hist1h1e |

| **7. CD8 T-cells III** | **8. NK cells** | **9. B-cells III** | **10. Monocyte/**  **macrophage** | **11. B-cells IV** | **12. Proliferating lymphocytes** |
| --- | --- | --- | --- | --- | --- |
| Ly6c2 | Gzma | Ighv8-5 | Lyz2 | Malat1 | Stmn1 |
| Ccl5 | Fcer1g | Iglv1 | Ifitm3 | Bank1 | Pclaf |
| Ctla2a | Tyrobp | AC133103.1 | Cebpb | Mef2c | Mki67 |
| Ctsw | Ncr1 | Zcwpw1 | Fcer1g | Bach2 | Top2a |
| Nkg7 | Klrb1c | Apoe | Ifitm2 | Ebf1 | Ube2c |
| Il2rb | Nkg7 | Mzb1 | Alox5ap | Macf1 | Birc5 |
| Klrd1 | Cd7 | E330020D12Rik | Tyrobp | Cd22 | Basp1 |
| Ms4a4b | AW112010 | Iglc1 | Lgals3 | Ralgps2 | Rrm2 |
| Ifitm10 | Ccl5 | Cyp11a1 | Ccl6 | Dmxl1 | Ccnb2 |
| Gzmm | Klrk1 | Ighv3-3 | Lst1 | Gm43305 | Mef2b |
| Klrk1 | Klra8 | 1810046K07Rik | Apoe | Gm26917 | Cks1b |
| Hopx | Prf1 | Fcrl5 | Ms4a6c | Pax5 | Cdca8 |
| Zyx | Klre1 | Hepacam2 | Msrb1 | Swap70 | Cks2 |
| Cd7 | Gzmb | Tcf4 | Csf1r | Lrrk2 | Lmnb1 |
| Il7r | Anxa2 | Gm15228 | Gngt2 | Myo1e | Hist1h1b |
| Klrc1 | Klra4 | C130026I21Rik | S100a4 | Etnk1 | Cenpa |
| Arl4c | Ccl4 | Camk2n1 | Ifitm6 | March1 | Hmgb2 |
| Xcl1 | Ctsw | Zbtb32 | Cybb | Bmp2k | Dut |
| Thy1 | Il2rb | Gm43305 | Plbd1 | Tmem131l | Hist1h2ae |
| Klra7 | Klra7 | Aldh2 | Tgfbi | Syk | H2afx |
| Cst7 | Ccl3 | Pkig | Cst3 | Fchsd2 | Hmgn2 |
| Hcst | Cma1 | Plac8 | Ctsb | Sorl1 | Hist1h2ap |
| Dnajc15 | Klrd1 | Vars | Gpx1 | Malt1 | H2afz |
| S100a6 | S1pr5 | Pdia4 | Psap | Eml4 | Tuba1b |
| Cd8b1 | Lgals1 | Napsa | Plac8 | Xist | Tubb5 |

| **13. CD4 T-cells III** | **14. DCs** | **15. B-cells V** | **16. B-cells VI** | **17. Neutrophils** | **18. pDCs** |
| --- | --- | --- | --- | --- | --- |
| Malat1 | Ccnd1 | Jchain | Hist1h1b | S100a9 | Siglech |
| Ptprc | Plbd1 | Slpi | Igkv8-28 | S100a8 | Cox6a2 |
| Prkch | Alox5ap | Mt1 | Hist1h2ae | Retnlg | Klk1 |
| Ikzf2 | Tmem176b | Derl3 | Hist1h2ab | Ngp | Ctsl |
| Itga4 | Ifitm3 | Creld2 | Hist1h2ak | Il1b | Mpeg1 |
| Mbnl1 | Pmaip1 | Edem1 | Igkv3-2 | Lcn2 | Alox5ap |
| Tnfaip3 | Ckb | Xbp1 | Pclaf | Ccl6 | Lair1 |
| Utrn | Cfp | Iglv3 | Hist1h1a | Slpi | Csf2rb2 |
| Kmt2a | Tbc1d4 | Igha | Hist1h1d | Csf3r | Smim5 |
| Maf | Marcks | Txndc5 | Ighv1-9 | Ifitm2 | Ccnd1 |
| Inpp4b | Tmem176a | Iglv1 | Hist1h2ap | Wfdc21 | Ccr9 |
| Macf1 | S100a4 | Iglc1 | Stmn1 | Alox5ap | Pltp |
| Lnpep | Tyrobp | Mzb1 | Hist1h1e | Hp | Grn |
| Ankrd11 | Spi1 | Igkc | Igkv12-46 | Ifitm1 | Atp1b1 |
| Cd5 | H2-DMb1 | Herpud1 | Hmgb2 | Cxcl2 | Tyrobp |
| Fryl | Gm2a | Slc3a2 | H2afz | Slc7a11 | Pld4 |
| Ahnak | Atox1 | Pdia4 | Hist1h4d | Mmp9 | Tcf4 |
| Hspa5 | Ifi30 | Ssr4 | Lgals1 | Cxcr2 | Bst2 |
| Itgb1 | Syngr2 | Hsp90b1 | Gapdh | Anxa1 | Rnase6 |
| Cd4 | Cst3 | Ighv6-6 | Hmgb1 | Msrb1 | Ly6c2 |
| Prrc2b | H2-Ab1 | Ighm | Tubb5 | Tyrobp | Irf8 |
| Itgal | H2-Eb1 | Fos | Tuba1b | Cebpb | Ctsb |
| Cd6 | H2-Aa | Jun | Actn4 | S100a6 | Plac8 |
| Malt1 | Rgs1 | Igkv3-4 | Slbp | S100a11 | Lgals1 |
| Gm26917 | Egr1 | Apoe | Mzb1 | Dusp1 | Psap |

**Supplemental Table S9. B-cell clustering top 25 differentially expressed genes**

| **1. FOB** | **2. MZB** | **3. ABC** | **4. Act. B** | **5. Tr. B** |
| --- | --- | --- | --- | --- |
| Fcer2a | Cr2 | AC133103.1 | Srm | H3f3a |
| Cd55 | Pik3r4 | Lgals1 | Hsp90ab1 | Vpreb3 |
| Shisa5 | Dtx1 | Zeb2 | Apex1 | Ly6d |
| Sell | Plac8 | Itgb1 | Nme1 | Cd79b |
| Jund | Rplp1 | Fcer1g | C1qbp | Iglc1 |
| Klf2 | Myof | Slpi | Ncl | Ms4a1 |
| Gmfg | Marcks | Tbx21 | Mif | Cd24a |
| Tsc22d3 | Rgs10 | C130026I21Rik | Eif4a1 | Ifi30 |
| Ighd | Dph5 | Adgre1 | Eif5a | Fam129c |
| Ypel3 | Rps12 | Fgl2 | Ppp1r14b | Ucp2 |
| Lmo2 | Cd1d1 | Spn | Fbl | Spib |
| Fchsd2 | Tm6sf1 | Adssl1 | Ranbp1 | Iglc2 |
| B4galnt1 | A930005H10Rik | Ahnak | Nme2 | Pafah1b3 |
| Serpinb1a | Tpst2 | Itm2c | Nhp2 | Klf2 |
| Ccr7 | Pdia4 | Lgals3 | Pa2g4 | Dnajc7 |
| Stk17b | Fcrl5 | Scimp | Npm1 | Hck |
| Ier2 | Ell3 | Fcgr2b | Hspe1 | Siglecg |
| Emp3 | Ly6a | Cd72 | Marcksl1 | Tnfrsf13c |
| Pxk | Ube2r2 | Anxa2 | Il4i1 | Ifi27l2a |
| Gimap3 | Myc | Pld4 | Hspd1 | Them6 |
| Pxdc1 | Cd9 | H2-Q7 | Ran | Cib1 |
| Gm8369 | Plaur | Gns | Ldha | Mcl1 |
| Add3 | Mzb1 | Zbtb20 | Bcl2a1b | Pten |
| S1pr4 | Pdcd2l | Apoe | Myc | Arl5c |
| Bmyc | Bmp2k | Igkv3-4 | Tuba1b | Gm42031 |

| **6. B1/Breg** | **7. pMB** | **8. GCB** | **9. PC** | **10. Ifn B** |
| --- | --- | --- | --- | --- |
| Ighv8-5 | Malat1 | Pclaf | Jchain | Ifit3 |
| Zcwpw1 | Ptprc | Basp1 | Derl3 | Ifit1 |
| AC133103.1 | Macf1 | Mef2b | Tent5c | Ifit3b |
| E330020D12Rik | Bach2 | Mki67 | Mt1 | Rsad2 |
| 1810046K07Rik | Cd22 | Top2a | Slpi | Usp18 |
| Cyp11a1 | Malt1 | Rgs13 | Edem1 | Oasl2 |
| Camk2n1 | Kmt2c | H1fx | Creld2 | Ifit2 |
| Ighv3-3 | Dmxl1 | Birc5 | Xbp1 | Isg15 |
| Gm15228 | Etnk1 | Aicda | H13 | Rtp4 |
| Zbtb32 | Dock10 | Stmn1 | Slc3a2 | Irf7 |
| Hepacam2 | Kmt2a | Eaf2 | Txndc5 | Slfn5 |
| S100a6 | Arhgap26 | Anxa2 | Igha | Isg20 |
| D10Wsu102e | Mycbp2 | Cks2 | Ssr4 | Zbp1 |
| C130026I21Rik | Ep300 | Lmnb1 | Igkc | Ifi213 |
| Fcrl5 | Sorl1 | Mcm3 | Hsp90b1 | Ifi47 |
| Iglv1 | Gm26917 | Hmces | Iglv1 | Ifi208 |
| Apoe | Gm43305 | Hmgb2 | Mzb1 | Bst2 |
| Zbtb20 | Lnpep | Txn1 | Pdia4 | Tor3a |
| Ahnak | Kat6a | H2afx | Ighg3 | Trim30a |
| Nfatc1 | Itga4 | Hmgn2 | Iglv3 | Phf11b |
| Plp2 | Tnks2 | Hist1h1b | Iglc1 | Stat1 |
| Plac8 | Sptbn1 | H2afz | Ighm | Irgm1 |
| Myc | Ankrd11 | Hist1h2ae | Fos | Lgals9 |
| Mzb1 | Birc6 | Tuba1b | Ighv6-6 | Ms4a4c |
| Vim | Xist | Hist1h2ap | Igkv3-4 | Ifi27l2a |

## 3. Supplemental Figures
